# NINJ1 plays a vital role in the release of neutrophil extracellular traps during acute lung injury

**DOI:** 10.1038/s41419-026-08995-5

**Published:** 2026-06-25

**Authors:** Wen-Jing Zhong, Yu-Biao Liu, Xin-Yu Yang, Meng-Rui Chen, Nan-Shi-Yu Yang, Chen-Yu Zhang, Jian-Bing Xiong, Wei-Feng Tang, Cha-Xiang Guan, Yan-Feng Zhang, Jia-Xi Duan, Yong Zhou

**Affiliations:** 1 Department of Geriatric Respiratory and Critical Care Medicine, Xiangya Hospital, Central South University, Changsha, Hunan China; 2 Department of Physiology, Xiangya School of Basic Medical Sciences, Central South University, Changsha, Hunan China; 3 National Experimental Teaching Demonstration Center for Medical Function, Central South University, Changsha, Hunan China; 4 Key Laboratory of General University of Hunan Province, Basic and Clinical Research in Major Respiratory Disease, Changsha, Hunan China; 5 National Clinical Research Center of Geriatric Disorders, Xiangya Hospital, Central South University, Changsha, Hunan China; 6 Department of Emergency, Xiangya Hospital, Central South University, Changsha, Hunan China; 7 Department of Pulmonary and Critical Care Medicine, The Second Xiangya Hospital, Central South University, Changsha, Hunan China; 8 School of Basic Medicine, Ningxia Medical University, Yinchuan, Ningxia China

**Keywords:** Respiration, Immune cell death

## Abstract

Excessive neutrophil extracellular traps (NETs) formation is a significant contributor to acute lung injury (ALI), making its inhibition a novel therapeutic avenue to improve outcomes. In this study, we revealed that a novel pore-forming protein ninjurin-1 (NINJ1) was highly expressed in pro-inflammatory neutrophil subpopulations during ALI, using public single-cell RNA sequencing and hotspot analysis. Furthermore, we demonstrated that the NINJ1 oligomerization was essential for the NET release in neutrophils from both acute respiratory distress syndrome (ARDS) patients and ALI mice. Genetic ablation of *Ninj1* in neutrophils abolished NET release, thereby attenuating pulmonary dysfunction and reducing ALI-related lethality. Mechanistically, we found that K45 and N60 are critical for NINJ1 oligomerization and subsequent NET release. In summary, our findings reveal a novel pore-forming protein-mediated mechanism for NET release and highlight NINJ1 as a potential therapeutic target for the treatment of ALI/ARDS.

**Figure:**
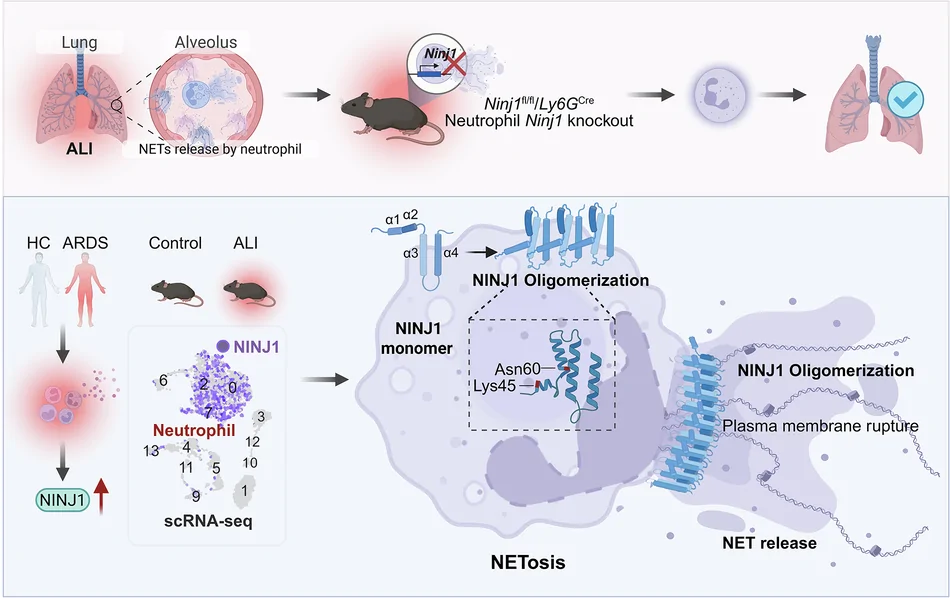
**Schematic illustration**. The novel pore-forming protein NINJ1 mediates the extrusion of NETs, thereby exacerbating pulmonary injury in ARDS/ALI. K45 and N60 are essential for NINJ1 oligomerization and subsequent NET release.

**Schematic illustration**. The novel pore-forming protein NINJ1 mediates the extrusion of NETs, thereby exacerbating pulmonary injury in ARDS/ALI. K45 and N60 are essential for NINJ1 oligomerization and subsequent NET release.

## Introduction

Acute lung injury (ALI) is characterized by acute hypoxemia and bilateral pulmonary edema, resulting from widespread inflammation and increased alveolocapillary permeability, and is associated with high mortality [1]. This condition is triggered by predisposing risk factors such as pneumonia, trauma, inhalation injury, thermal injuries, extrapulmonary sepsis, pancreatitis, massive transfusion, or shock [2]. Although the 2012 Berlin definition reclassified ALI as mild acute respiratory distress syndrome (ARDS), the term ALI continues to be widely used in preclinical research [3]. Advances in critical care management, particularly the adoption of lung-protective ventilation strategies, have markedly improved outcomes in ARDS patients over the past decade [4]. Nevertheless, mortality rates remain unacceptably high, highlighting an urgent need for effective pharmacotherapeutic interventions.

Neutrophils play a pivotal role in the pathogenesis of ARDS primarily through the formation of neutrophil extracellular traps (NETs), which have emerged as a promising therapeutic target [5]. NETs are DNA fragments with cytoplasm proteins released from neutrophils, which are involved in the pathogenesis of ALI/ARDS and represent a promising therapeutic target [6]. NETs are web-like structures composed of DNA filaments decorated with cytoplasmic and granular proteins, which are released by neutrophils in response to inflammatory activation. These structures, containing components such as citrullinated histone H3 (citH3), myeloperoxidase (MPO), neutrophil elastase (NE), and cell-free double-stranded DNA (dsDNA), contribute significantly to the pro-inflammatory response and are implicated in the pathogenesis of ALI/ARDS [7]. The process by which neutrophils form NETs is a specialized form of lytic cell death termed NETosis. Elevated levels of NETs in peripheral blood, as measured by plasma MPO-dsDNA complexes, are positively associated with disease severity and inversely correlated with the ratio of partial pressure of arterial oxygen to fraction of inspired oxygen (PaO_2_/FiO_2_ ratio) in patients with ARDS [8]. Our previous study also suggested that NETs trigger alveolar epithelial cell death *via* caspase-independent necroptosis, which is initiated by the sensing of NET-derived dsDNA through the cyclic GMP-AMP synthase stimulator of the interferon gene (cGAS/STING) pathway during ALI [9]. Besides, NETs trigger the production of reactive oxygen species (ROS) and activation of the nucleotide-binding oligomerization domain-like receptor protein 3 (NLRP3) inflammasome, mediating macrophage pyroptosis and triggering endothelial cell ferroptosis in ALI [10]. These studies indicate that the DNA scaffold of NETs serves as a damage-associated molecular pattern (DAMP), triggering diffuse alveolar damage (DAD), inflammatory cascades, and vascular endothelial injury, thereby disrupting the air-blood barrier. Therefore, targeting NET release is critical for treating ALI/ARDS.

NET release occurs through a stepwise and highly orchestrated process, involving sequential stages of cytoskeletal reorganization, plasma membrane vesiculation, chromatin decondensation, nuclear envelope disintegration, and culminating in plasma membrane rupture (PMR) [11]. The NETs scaffold is composed of chromatin filaments (approximately 10-15 nm in diameter) studded with granular and cytoplasmic proteins (forming clusters of 20-50 nm), which assemble into a porous meshwork ranging from 50 to 200 nm. PMR serves as a prerequisite for releasing the DNA-based NET structures [12]. Traditionally, lytic cell death-induced PMR was viewed as a passive process of osmotic lysis driven by pore-forming proteins [13, 14], most notably Gasdermin D (GSDMD) and mixed-lineage kinase domain-like protein (MLKL), which mediate PMR during pyroptosis and necroptosis respectively [15, 16]. However, the conventional pore-forming proteins may not be the primary drivers of NET release. Evidence shows that NET release can occur independently of MLKL [17], and the small diameter of GSDMD pores (approximately 18 nm inner diameter) is insufficient to release the large DAMPs including NETs [18]. This mechanistic gap was recently addressed by the identification of ninjurin-1 (NINJ1) [19]. Kayagaki et al. identified NINJ1 as an active executioner of PMR, demonstrating its essential role in pyroptotic release of large DAMPs, such as lactate dehydrogenase (LDH, 140 kDa) [20]. This 16 kDa transmembrane protein was initially identified as an adhesion molecule expressed following nerve injury [21]. Its subsequent recognition as a key mediator of PMR in pyroptosis represents a paradigm-shifting discovery. Kayagaki and colleagues showed that NINJ1 oligomerizes into large filaments in the induction of PMR [22]. David et al. suggest that NINJ1 oligomerizes into irregular rings to execute PMR by jettisoning membranes like a “cookie-cutter” [23]. However, the precise details of NINJ1’s role in NET release and the molecular mechanism underlying NINJ1 oligomerization are still not precise.

Here we investigated whether the PMR executor NINJ1 induced NET release and exacerbated pulmonary injury in ALI. Single-cell RNA sequencing and Hotspot analysis identified NINJ1 upregulation in neutrophils and its association with NETosis. Genetic depletion of *Ninj1* in neutrophils attenuated NET release, reduced lung damage, and mitigated histopathology in ALI mice. Mechanistically, neutrophils from ARDS patients exhibited NINJ1 oligomerization on membranes alongside NET structures. K45 and N60 are critical for NINJ1 oligomerization and subsequent NET release. Collectively, our findings unveil a novel role for NINJ1 oligomerization in NET-mediated exacerbation against ALI.

## Materials and methods

### Ethics statement

All animal experiments described here complied with the guidelines of the Committee on the Use and Care of Animals of Central South University (Changsha, China) and were approved by the IRB of the Xiangya School of Basic Medical Science at Central South University (2023-KT107). Human neutrophils were obtained from patients who met the Berlin definition of ARDS at the Intensive Care Unit of Xiangya Hospital, Central South University (Changsha, China). All patients signed informed consent for their samples to be used for research, and the Clinical Medical Ethics Committee of Xiangya Hospital, Central South University, approved the study (2024030484, Changsha, China).

### Mice and induction of ALI

Adult male C57BL/6J mice (8–10 weeks of age, 20–25 g) were purchased from Hunan SJA Laboratory Animal Co., Ltd (Hunan, China). The mice were maintained in a specific pathogen-free environment at the Central South University Animal Facility. Mice were randomly allocated to experimental groups using a computer-generated randomization schedule. To establish the ALI model, C57BL/6J mice received an intratracheal instillation of lipopolysaccharide (LPS, 5 mg/kg in 50 μL saline; derived from E. coli O111:B4, Sigma-Aldrich) and were euthanized 12 h later. Mice in the Control group were administered an equal volume of saline *via* the same route. Mice with unsuccessful intratracheal instillation (immediate reflux of the instilled solution, or death during or immediately after the procedure) were excluded from the analysis, based on pre‑established experimental criteria.

The conditional *Ninj1* knockout mouse model (*Ninj1*^fl/fl^ on a C57BL/6J background) was generated by Shanghai Model Organisms Center (Cat. No. NM-CKO-200039, China) using CRISPR/Cas9-mediated genome editing. Briefly, a circular donor vector harboring *loxP* sites within intron 1 was constructed. Cas9 mRNA, sgRNAs, and the donor vector were co-microinjected into the cytoplasm of fertilized one-cell C57BL/6J embryos, yielding chimeric founders that were subsequently bred to wild-type C57BL/6J mice to obtain germline-transmitted *Ninj1*^fl/+^ offspring. The *Ninj1*^fl/fl^ mouse was then crossed with a mouse carrying the Cre recombinase selectively in neutrophils under the control of the endogenous lymphocyte antigen 6 complex (*Ly6G*) promoter/enhancer (official name: *Ly6G*-Cre-2A-tdTomato mice, Cat. No. NM-KI-200219, Shanghai Model Organisms Center, China), to generate the neutrophil conditional *Ninj1* knockout (*Ninj1*^fl/fl^*/Ly6G*^Cre^, referred as *Ninj1*^ΔNeu^ in the manuscript) mice. For all experiments, *Ly6G*-Cre-*Ninj1*^fl/fl^ (referred to as *Ninj1*^WT^ in the manuscript) littermates were used as controls for *Ninj1*^ΔNeu^ mice. Mice were maintained under specific pathogen-free conditions with a 12-hour light/dark cycle and provided food and water ad libitum.

### Collection of bronchoalveolar lavage fluid (BALF)

BALF was obtained by performing intratracheal instillation with 0.8 mL of ice-cold saline, a process repeated three times in accordance with established methodology [24]. Following collection, the BALF was centrifuged at 500 × g for 5 min at 4 °C. The resulting cell pellet was resuspended in 0.5 mL of PBS. The total protein concentration in the BALF supernatant, which serves as a marker of alveolocapillary barrier integrity, was quantified using a commercially available bicinchoninic acid (BCA) assay kit (Thermo Fisher Scientific).

### Real-time quantitative polymerase chain reaction (Real-time PCR)

Total RNA was isolated from both tissue samples and cultured cells using RNAiso Plus reagent (Takara, Japan) according to our prior works [25, 26]. RNA purity and concentration were verified by spectrophotometry (A260/A280 ratio > 1.8). First-strand cDNA synthesis was performed using the PrimeScript RT reagent kit (Takara) with oligo (dT) primers. Quantitative PCR analysis was conducted on a CFX96 Touch^TM^ system (Bio-Rad Laboratories, CA, USA) using SYBR Green Master Mix (Applied Biosystems) at the Top-Notch Innovation Base of Basic Medicine (Central South University). Gene expression levels were normalized to β-actin and calculated using the comparative 2^−ΔΔCt^ method. All primer sequences were designed using NCBI Primer-BLAST and are detailed in Table 1.

**Table 1:** Sequences of the primers used to quantify gene expression.

Gene	Forward primer (5′–3′)	Reverse primer (5′–3′)
*Mcp1*	GTCCCTGTCATGCTTCTGG	GCGTTAACTGCATCTGGCT
*pro-IL-1β*	CAGGCAGGCAGTATCACTCA	AGCTCATATGGGTCCGACAG
*Trem-1*	CTGTGCGTGTTCTTTGTC	CTTCCCGTCTGGTAGTCT
*Tnf-α*	AGCCCCCAGTCTGTATCCTT	CTCCCTTTGCAGAACTCAGG
*Nlrp3*	TACGGCCGTCTACGTCTTCT	CGCAGATCACACTCCTCAAA
*Ninj1*	GAGTCGGGCACTGAGGAGTAT	CGCTCTTCTTGTTGGCATAATGG
*β-actin*	TTCCAGCCTTCCTTCTTG	GGAGCCAGAGCA GTAATC

### Western blot

Protein extracted from lung tissue, neutrophils, or supernatants was subjected to Western blot analysis following established protocols from our prior work [24, 27]. In brief, lung tissue or neutrophil samples were lysed using RIPA buffer (Solarbio, Beijing, China) supplemented with protease and phosphatase inhibitors. Total protein content was quantified *via* the BCA method. To concentrate proteins from supernatants, 700 µL supernatant was combined with 700 µL 100% methanol and 175 µL chloroform, followed by vigorous vortexing for 30 s. The mixture was centrifuged at 13,000 × g for 5 min at 4 °C. After discarding the upper aqueous phase, 700 µL of 100% methanol was added to the interphase and organic layer, and the sample was recentrifuged under the same conditions. The resulting pellet was air-dried and resuspended in 20 µL of 10% SDS. Subsequently, 4 µL of 5 × SDS-PAGE loading buffer (Beyotime, Jiangsu, China) was added, and the sample was denatured at 95 °C for 10 min. Subsequently, equal quantities of protein (30 μg per lane) or all proteins from supernatants were separated by electrophoresis on 12% SDS‑polyacrylamide gels. Then they transferred onto polyvinylidene fluoride (PVDF) membranes (Millipore, USA). The membranes were blocked with 5% non-fat milk or bovine serum albumin (BSA) for 1.5 h, probed with primary antibodies at 4 °C overnight, and incubated with horseradish peroxidase (HRP)-conjugated secondary antibodies for one hour at room temperature. Band intensities were quantified using Image Lab Analyzer software (Bio-Rad) and normalized to the loading control (α-tubulin or β-actin). All normalized values were then expressed relative to the Control. The NINJ1 antibody used has been validated in multiple studies [28, 29]. Antibody details are provided in Table 2.

**Table 2:** Antibody sources and dilutions.

Antibodies	Source	Catalog	Dilution ratio
**Primary antibodies for Western blot**			
Anti-NINJ1 monoclonal antibody	Santa Cruz	sc-136295	1:2000
Anti-NINJ1 monoclonal antibody	Invitrogen	PA5-95755	1:2000
Anti-MPO polyclonal antibody	Proteintech	66177-1-Ig	1:2000
Anti-citH3 polyclonal antibody	Abcam	Ab5013	1:2000
Rabbit-anti-ELANE antibody	Abclonal	A8953	1:2000
Anti-PAD4 polyclonal antibody	Santa Cruz	sc-365369	1:2000
Anti-IL-1β polyclonal antibody	R&D	AF-401-NA	1:2500
Anti-NLRP3 polyclonal antibody	CST	15101	1:2000
Anti-β-actin monoclonal antibody	ABclonal	AC026	1:5000
Anti-α-tubulin monoclonal antibody	Servicebio	GB11200	1:10,000
**Primary antibodies for immunofluorescence**			
Anti-NINJ1 monoclonal antibody	Mybiosource	MBS249960	1:100
Anti-NINJ1 monoclonal antibody	Invitrogen	PA5-95755	1:100
**Secondary antibody**			
HRP-conjugated goat anti-rabbit IgG	SAB	#L3012-2	1:5000
Goat anti-Mouse IgG	SAB	L3032	1:5000
Rabbit anti-Goat IgG	SAB	L3042-2	1:5000
FITC-conjugated Goat anti-Rabbit IgG (H + L)	Abclonal	AS011	1:400
TRITC-conjugated Goat anti-Mouse IgG	Abclonal	AS026	1:400

### Native and SDS-PAGE of NINJ1

Neutrophils were lysed with native-PAGE lysis buffer (150 mM NaCl, 1% Triton X-100, 50 mM Tris, pH 7.5, and 1×Complete PMSF, Solarbio, R0030). Following centrifugation at 20,800 × g for 30 min, lysates were mixed with 5×Native Sample Loading Buffer (Sangon Biotech, Shanghai, China, C506032) and resolved using Native PAGE^TM^ 3–12% Bis-Tris gels (BBI Life Sciences Corporation, C631101-0100). For SDS-PAGE analysis, cells were rinsed with phosphate-buffered saline (PBS) and lysed in RIPA buffer (Beyotime) supplemented with protease inhibitor cocktail (Roche, Mannheim, Germany). Equivalent quantities of native protein were separated under non-reducing conditions using pre-cast 3–12% Bis-Tris gels with Tris-Glycine native running buffer (pH 8.8, Sangon Biotech) at 4 °C. Equivalent quantities of denatured protein were separated under reducing conditions on a 12% gradient polyacrylamide gel at room temperature. Following electrophoresis, proteins were transferred to PVDF membranes and probed using standard immunoblotting procedures.

### Immunofluorescence staining (IF)

IF staining was carried out on 3-μm paraffin-embedded lung sections or fixed cultured cells. Sections or cells were rinsed with PBS and fixed using 4% paraformaldehyde for 15 min at room temperature. Permeabilization was carried out with 0.1% Tween-20. Nonspecific binding sites were subsequently blocked by incubating the cells for one hour at room temperature in a solution of PBS containing 10% BSA. Finally, samples were incubated overnight at 4 °C with the following primary antibodies: anti-NINJ1 (mouse, Mybiosource, 1:100), anti-MPO (rabbit, 1:200, Proteintech), anti-citH3 (rabbit, 1:200, Abcam), and anti-GM130 (rabbit, 1:100, Proteintech). After washing, samples were treated with species-appropriate secondary antibodies conjugated to fluorophores for one hour at room temperature, protected from light. Nuclei were counterstained with DAPI (Solarbio). Images were captured using a laser scanning confocal microscope (Leica SP8, Germany).

### Lung histology and inflammatory injury score

At 12 h post-LPS administration, mice were euthanized, and the left lung lobe was fixed in 4% neutral buffered formaldehyde at 4 °C. Tissue sections (4 μm) were prepared and stained with hematoxylin and eosin (H&E; Solarbio). Whole-slide imaging was performed using a Pannoramic Scan system (3Dhistech, Budapest, Hungary). A rigorous histopathological analysis was conducted in accordance with our previous studies by three independent pathologists (blinded to treatment groups) who employed a standardized scoring matrix (0 = normal; 4 = severe) to assess: (a) neutrophil-dominant mixed alveolar inflammation, (b) pseudostratified bronchoalveolar hyperplasia, (c) multifocal hemorrhagic foci, (d) eosinophilic lipoproteinosis, and (e) fibrinous hyaline membranes. Interobserver variability was minimized through consensus training.

### Micro computed tomography (micro-CT)

Micro-CT was performed using a small animal live micro-CT system (Quantum GX, PerkinElmer, USA; instrument ID: 00130128). Before scanning, mice were anesthetized to ensure immobility during image acquisition. The Quantum GX system uses an X-ray source and detector that rotate 360° continuously around the animal to acquire a series of two-dimensional (2D) projection images. For in vivo imaging, a field of view of 36 mm or 72 mm was used to cover the whole mouse body. A standard fast scan was completed in 8 seconds with typical parameters set at 90 kV tube voltage and 72–88 µA tube current. After image reconstruction, three-dimensional (3D) data were processed using the integrated Quantum GX image analysis software. Lung parameters, including lung volume and mean lung density, were quantified to assess lung injury severity.

### Measurement of LDH

LDH activity was assessed with a commercial assay kit (Jiancheng Bioengineering Institute, Nanjing, China) according to the manufacturer’s instructions. Quantification was based on absorbance measurements at 450 nm.

### Enzyme-linked immunosorbent assay (ELISA)

Levels of tumor necrosis factor-alpha (TNF-α) in both lung tissue homogenates and serum were quantified using commercially available ELISA kits (Invitrogen; Thermo Fisher Scientific) following the supplier’s guidelines. Similarly, MPO-dsDNA and citH3-dsDNA complexes were assessed with ELISA kits (JONLNBIO, China) as per the manufacturer’s instructions. Absorbance readings taken at 450 nm were converted to concentration values by comparison with a standard curve. Cytokine measurements were normalized to total protein concentration, which was determined using a BCA protein assay kit.

### The survival rate of ALI mice

Mice were administered LPS (25 mg/kg, *i.t*.) to induce ALI. Mortality was monitored every 6 h over 84 h, and survival rates were plotted as a Kaplan‑Meier survival curve (*n* = 16 per group).

### Single-cell transcriptome sequencing data analysis

Single-cell RNA sequencing data were processed with Seurat (v5) [30]. For the GSE224938, GSE36809070 dataset, cells were filtered based on the following criteria: exclusion of those with gene counts greater than 7000 or fewer than 700; removal of cells where mitochondrial UMI counts exceeded 10% or erythrocyte-derived UMI counts comprised more than 20%. Normalization was carried out using the “NormalizeData” function, followed by data scaling *via* “ScaleData”. The 2000 most highly variable genes were identified using “FindVariableFeatures”. Dimensionality reduction was performed using principal component analysis (PCA) based on these variable genes, with a resolution parameter of 0.4. Batch effects were mitigated through dataset integration across conditions employing the “RunHarmony” function. Potential marker genes were detected using “FindAllMarkers”. Cell type annotation was performed by referencing canonical marker genes. Cellular clusters were visualized using Uniform Manifold Approximation and Projection (UMAP). Neutrophil subpopulations were clustered into four distinct subsets using the FindClusters function.

### Pseudotime analysis

To delineate cellular differentiation trajectories, pseudotime analysis was performed using Monocle2 (v2.26.0) [31]. Genes with a mean expression level above 0.1 were initially selected for inclusion. Subsequently, the “differentialGeneTest” function was applied to filter out genes unsuitable for cell ordering, based on a q-value cutoff of < 0.01. Dimensionality reduction was carried out *via* the “reduceDimension” method with the DDRTree algorithm. All analyses were conducted using default parameters. Final cell ordering was determined using the “order Cells” function.

### Identification of Hotspot gene modules in ALI data

We employed Hotspot (v.0.9.1)^20^ to detect context-specific gene modules within a single-cell RNA-seq dataset (GSE224938). This algorithm utilizes a user-defined cell-cell similarity metric to construct a *k*-nearest neighbor (*k*-NN) graph, upon which it assesses pairwise local correlations between genes within defined cellular neighborhoods. Genes exhibiting significant local autocorrelation are identified, an approach particularly suited to scRNA-seq data, as it remains robust to technical artifacts such as gene dropouts. The resulting gene-gene affinity matrix is subsequently clustered to define discrete gene modules. Unlike global correlation measures (e.g., Pearson’s correlation), which assume uniform feature relationships across the entire dataset, Hotspot evaluates dependencies within local contexts of the *k*-NN graph, thereby capturing spatially or biologically restricted co-expression patterns.

Employing non-negative matrix factorization (NMF) to uncover gene programs has been reported in prior studies [32]. However, as a linear decomposition technique, NMF necessitates a coherent and comprehensive representation of the full dataset, and can exhibit sensitivity to technical artifacts such as batch variation. In contrast, Hotspot identifies gene modules through the analysis of gene-gene covariance, which more effectively captures cooperative gene sets underlying shared biological processes and demonstrates enhanced robustness to batch effects [33]-making it particularly suitable for analyzing heterogeneous neutrophil populations. A distinctive feature of Hotspot is its ability to detect covariances that are spatially restricted to specific cell subpopulations or that display nonlinear, graded patterns across regions of the data manifold. This is especially relevant in ALI, where adaptation to diverse microenvironments-such as disseminating infection-may drive context-specific changes in gene co-expression. Furthermore, Hotspot accommodates both gene pleiotropy and rare cell states, which are salient features of ALI inflammation pathogenesis. Accordingly, we applied Hotspot to dissect the sources of inter-and intra-neutrophil phenotypic heterogeneity in our data, in addition to identifying global, manifold-defining gene modules.

To implement Hotspot, we first isolated neutrophils from the dataset (*n* = 856). Genes expressed in fewer than 1% of neutrophils or with zero total expression were filtered out, resulting in a final set of 6578 genes for downstream analysis. We next identified a subset of genes exhibiting significant spatial autocorrelation within this latent PC space by executing Hotspot with the depth-adjusted negative binomial (danb) observation model and a neighborhood size of 30. The danb model provides a null background distribution for normalizing expression counts, thereby reducing false positives arising from local correlations in cellular library size [34].

We retained 1284 genes exhibiting a false discovery rate (FDR) < 0.001 for subsequent local correlation analysis and module clustering (described below). Using the create_-_modules function in Hotspot, with default parameters aside from a minimum gene threshold of 30 and core-only=True, we identified an initial set of 10 co-expression modules. The Hotspot module definition is exhaustive and mutually exclusive, with each of the 1284 spatially variable genes assigned to exactly one module based on its local correlation pattern. Gene assignments for these modules are provided in Suppl. Table 1. Among these ten modules, module 8 emerged as the primary focus, as it contained NINJ1 and was significantly enriched for genes in the NETosis pathway (KEGG: mmu04613). Consistent with the per‑spot NETosis score enrichment shown in Fig. 2D, the vast majority of NETosis‑related genes segregated into module 8 (annotated in the updated Fig. 2A heatmap). To quantify NETosis activity at the transcriptional level, a NETosis score was calculated using Seurat’s AddModuleScore function, which calculates the average expression of the KEGG NETosis gene set, subtracted by control gene sets. This score reflects the aggregate NET‑formation transcriptional program. The updated figure and accompanying source data file (Suppl. file 1 modules_filtered_p0.001_top gene30) clearly demonstrate this co‑localization. Hotspot is implemented as an open-source Python package and is available for use at http://www.github.com/Yoseflab/Hotspot.

### Recruitment of healthy controls (HCs) and clinical ARDS patients

Patients diagnosed with ARDS according to the Berlin definition were included in this study. Between March 2024 and March 2025, blood samples were collected from 12 patients with ARDS admitted to the intensive care unit (ICU) of Xiangya Hospital, Central South University, Changsha, China. ARDS etiologies comprised sepsis, pneumonia and post-surgical conditions. All samples were obtained after ARDS diagnosis was confirmed (PaO₂/FiO₂ ≤ 300). Exclusion criteria included pregnancy or lactation, age under 18 years, active hematologic malignancy or solid tumor cancer, end-stage organ disease, and autoimmune disorders. Age- and sex-matched healthy controls (*n* = 12) were recruited from the health examination center of the same hospital. These controls had no history of acute or chronic inflammatory diseases or recent surgery. The following demographic and clinical parameters were extracted from electronic medical records (Table 3): age, sex, body mass index (BMI), PaO_2_/FiO_2_ ratio, Sequential Organ Failure Assessment (SOFA) score, Acute Physiology and Chronic Health Evaluation II (APACHE II) score, white blood cell count (WBC), neutrophil count (NEUT), C-reactive protein (CRP), and procalcitonin (PCT). Data on respiratory support, infection source, corticosteroid use, and anticoagulation were also collected. The individual-level raw data for all participants have been provided as Suppl. File 2 clinical data.

**Table 3:** Demographic and clinical characteristics of the ARDS patients.

Characteristic	ARDS (*n* = 12)
**Demographics**	
Age, years, mean (SD)	68.42 (9.69)
Male, *n* (%)	8 (67)
BMI, kg/m^2^, mean (SD)	22.25 (4.32)
**Clinical severity**	
PaO_2_/FiO_2_, median (IQR)	165 (122–222.5)
SOFA score, median (IQR)	5 (4–7.5)
APACHE II score, median (IQR)	17 (12–21.5)
**Respiratory support,** ***n*** **(%)**	
Invasive mechanical ventilation	7 (58.3)
Noninvasive ventilation	3 (25.0)
High-flow nasal cannula	2 (16.7)
**Infection status,** ***n*** **(%)**	
Pulmonary	8 (66.7)
Extrapulmonary	2 (16.7)
No documented infection	2 (16.7)
**Laboratory finding**	
WBC, 10^9^/L, median (IQR)	13.0 (10.15–18)
NEUT, 10^9^/L, median (IQR)	11.2 (8.5–16.35)
CRP, mg/L, median (IQR)	130.5 (64.25–197.5)
PCT, ng/mL, median (IQR)	0.56 (0.36–1.5)
**Medications,** ***n*** **(%)**	
Corticosteroid use	2 (16.7)
Anticoagulation therapy	2 (16.7)

Data are presented as median (interquartile range, IQR) for non-normally distributed variables.

### Neutrophil isolation, culture and NETosis induction

Neutrophils were isolated from the bone marrow of 8–10-week-old male C57BL/6J mice using a combination of immunomagnetic separation techniques. Briefly, femurs and tibias were dissected, and bone marrow cells were flushed out with cold PBS using a 26-gauge needle. After lysing red blood cells with red blood cell lysis buffer (Solarbio), the resulting hematopoietic cells were incubated with an Anti-Ly-6G MicroBeads UltraPure (Miltenyi Biotec) and sorted using an LS column and MACS MultiStand (Miltenyi Biotec) cell sorter. For the magnetic isolation procedure, bone marrow cells were labeled with Anti-Ly-6G MicroBeads UltraPure (Miltenyi Biotec). Ly6G⁺ cells were subsequently isolated using an LS column and MACS MultiStand (Miltenyi Biotec) cell sorter, in accordance with the manufacturer’s protocols. The purified neutrophil populations were resuspended in complete RPMI-1640 medium (Gibco) containing 10% bovine serum (BCS) and were maintained under standard culture conditions before subsequent experiments.

Peripheral blood samples were collected from HCs following written informed consent, with ethical approval granted by the IRB of the Xiangya School of Basic Medical Science at Central South University (approval reference number 2024030484) in compliance with Helsinki Declaration guidelines. Whole blood was mixed with 0.9% sodium chloride containing 2.0% Dextran T-500 (Solarbio) at a 1.5:1 ratio and incubated for 30 min at room temperature to sediment erythrocytes. The leukocyte-rich supernatant was then carefully layered onto a discontinuous Percoll (Cytiva) gradient prepared by sequentially overlaying 2 mL of 45%, 62%, and 75% Percoll solutions in a 15 mL conical tube using a sterile serological pipette. After centrifugation at 250 × g for 30 min with the brake disabled, peripheral blood neutrophils were collected from the interface between the 62% and 75% Percoll layers. The cells were washed with PBS, centrifuged at 250 × g for 8 min, and resuspended in RPMI 1640 medium supplemented with 10% BCS. Cell count and purity were determined using flow cytometry before subsequent experiments.

For NETosis induction, primary neutrophils were treated with 50 nM PMA or 100 ng/mL LPS for 3 h. To estimate the role of pore-forming proteins in NET release, mouse primary neutrophils were pre‑treated for 30 min with GSDMD inhibitor (Disulfiram, 5 μM, TargetMol, China), or RIPK3 inhibitor (GSK872, 1 μM, Medchem Express, USA) before PMA (100 nM) stimulation.

### Differentiated cell line (dHL‑60)

The human promyelocytic leukemia cell line dHL-60 was acquired from the American Type Culture Collection (RRID: CVCL_HL60). Cells were maintained at 37 °C under a 5% CO_2_ atmosphere in RPMI-1640 medium containing L-glutamine and 25 mM HEPES, and supplemented with 10% heat-inactivated BCS and 1% penicillin-streptomycin. To induce neutrophilic differentiation, dHL-60 cells were treated with 1 μM all-trans retinoic acid (ATRA) plus 1% dimethyl sulfoxide (DMSO) for 6 days. Differentiation efficiency was evaluated by Wright-Giemsa staining (Fig. S8). For NETosis induction, dHL‑60 cells were treated with 100 nM phorbol 12‑myristate 13‑acetate (PMA) for 3 h.

### Plasmids and Transfection (applied to dHL‑60 cells)

All expression plasmids were commercially sourced from Sangon Biotech. Site-directed mutagenesis was performed to generate the N60A variant of NINJ1. Both the wild-type and mutant coding sequences were amplified by PCR using primers engineered to incorporate 5′ Nhe I and 3′ Hind III restriction sites. The purified amplicons and the pcDNA3.1-3×FLAG vector were subjected to double digestion with the corresponding restriction endonucleases. The resulting fragments were purified and ligated into the linearized vector backbone using T4 DNA ligase to produce C-terminally 3×FLAG-tagged recombinant constructs.

For transient transfection, Lipofectamine^TM^ 3000 (Invitrogen) was used according to the manufacturer’s protocol. Briefly, differentiated dHL-60 cells, which had been cultured in differentiation medium for 72 h, were used for transfection. Before transfection, the plasmid DNA and Lipofectamine 3000 reagent were separately diluted in serum-free medium, combined at an appropriate ratio, and incubated for 20 min at room temperature to allow complex formation. The resulting transfection mixture was then added dropwise to the cells. Following gentle agitation to ensure uniform distribution, the medium was replaced with complete growth medium containing serum after 4–6 h. Cells were cultured for an additional 72 h before downstream analysis or functional assays.

### Flow cytometry

Peripheral blood neutrophils were isolated from ARDS patients and Healthy Controls. For surface staining, cells (1 × 10^6^) were incubated with anti-human NINJ1 (BD Pharmingen, San Jose, CA, USA, 610777) for 30 min at 4 °C in the dark. For intracellular staining of NINJ1, cells were first fixed with 4% paraformaldehyde, followed by incubation with anti-NINJ1 antibody and FITC secondary antibody. After washing, stained cells were resuspended in PBS containing 1% BSA and analyzed on a flow cytometer (BD Biosciences). Data were acquired for at least 10,000 events per sample, and the median fluorescence intensity (MFI) or percentage of positive cells was determined using FlowJo software (version 10, Tree Star Inc., San Jose, CA, USA). Dead cells were excluded based on forward/side scatter or by using a viability dye (PI).

### RNA sequencing and analysis

Total RNA isolation was performed with RNAiso Plus reagent (Takara) and sequenced on an Illumina NovaSeq 6000 platform (Majorbio, China). Raw sequencing data were obtained in FASTQ format, and an initial quality assessment was conducted with FASTQC. Low-quality reads were filtered out using FASTX-Toolkit. The remaining high-quality reads were aligned to the human reference genome (GRCh38) using TopHat, with gene annotation provided *via* the “-G” option using a human mRNA GTF file. Read counts per gene were generated with HTSeq. Differential gene expression analysis was performed using the DESeq2 package in R, comparing the Control and PMA groups. Genes with an adjusted *P*-value of less than 0.05 were considered statistically significantly differentially expressed. Bioinformatics analyses were carried out on the Majorbio Cloud Platform (Majorbio I-Sanger Cloud Platform, www.majorbio.com).

### Assay of PMR

Cells were collected and washed twice with HBSS for 5 min each. The cells were resuspended at a density of 1 × 10^6^/mL and treated with 1 mL of FM 4–64 working solution (MCE, 5 μM) for 30 min at room temperature. Following incubation, the samples were centrifuged at 400 × g for 4 min, and the supernatant was aspirated. The pellet was washed twice with HBSS and subsequently stained with DAPI. Finally, cells were resuspended in 1 mL of serum-free medium and examined by a laser scanning confocal microscope.

### Quantification of dsDNA

To quantify circulating NETs, levels of dsDNA in plasma, BALF, and cell supernatant were measured using the PicoGreen® dsDNA Quantification Kit (Solarbio). Whole blood collected from mice was transferred into 1.5 mL centrifuge tubes containing EDTA-Na as an anticoagulant. Plasma BALF and cell supernatant were separated by centrifugation at 3000×rpm for 15 min at 4 °C. The concentration of dsDNA in the plasma was then quantified using the PicoGreen® dsDNA Quantification Kit, following the manufacturer’s instructions.

### Scanning electron microscopy

For SEM analysis, the samples were primarily fixed in 2.5% glutaraldehyde in 0.1 M phosphate buffer (pH 7.4) for a minimum of 4 h at 4 °C. Following three washes with cold phosphate buffer, the samples were post-fixed in 1% osmium tetroxide for 1–2 h at room temperature. Subsequently, the samples were dehydrated through a graded series of ethanol solutions, with each step lasting for 15 min. Critical-point drying was then performed using liquid CO_2_ to preserve the ultrastructural integrity. The dried samples were mounted on aluminum stubs and sputter-coated with a thin layer of gold-palladium to enhance electrical conductivity. Finally, the specimens were examined under a Zeiss Sigma 300 scanning electron microscope, and representative images were captured.

### Statistical analysis

A minimum of three independent biological replicates was performed for each experiment. Data are expressed as mean ± standard deviation. Statistical analyses and graphical representations were conducted using GraphPad Prism 9 (San Diego, CA, USA). The data were tested for normality using the Shapiro-Wilk test. Normally distributed data are presented as mean ± standard deviation (SD), and non-normally distributed data as median with interquartile range (IQR). Comparisons between two groups were performed using an unpaired Student’s *t*-test, while differences among multiple groups were assessed by one-way ANOVA followed by Tukey’s or Dunnett’s post hoc test. A *P*-value below 0.05 was considered statistically significant.

## Results

### NINJ1 is enriched in neutrophils during ALI

**Fig. 1:**
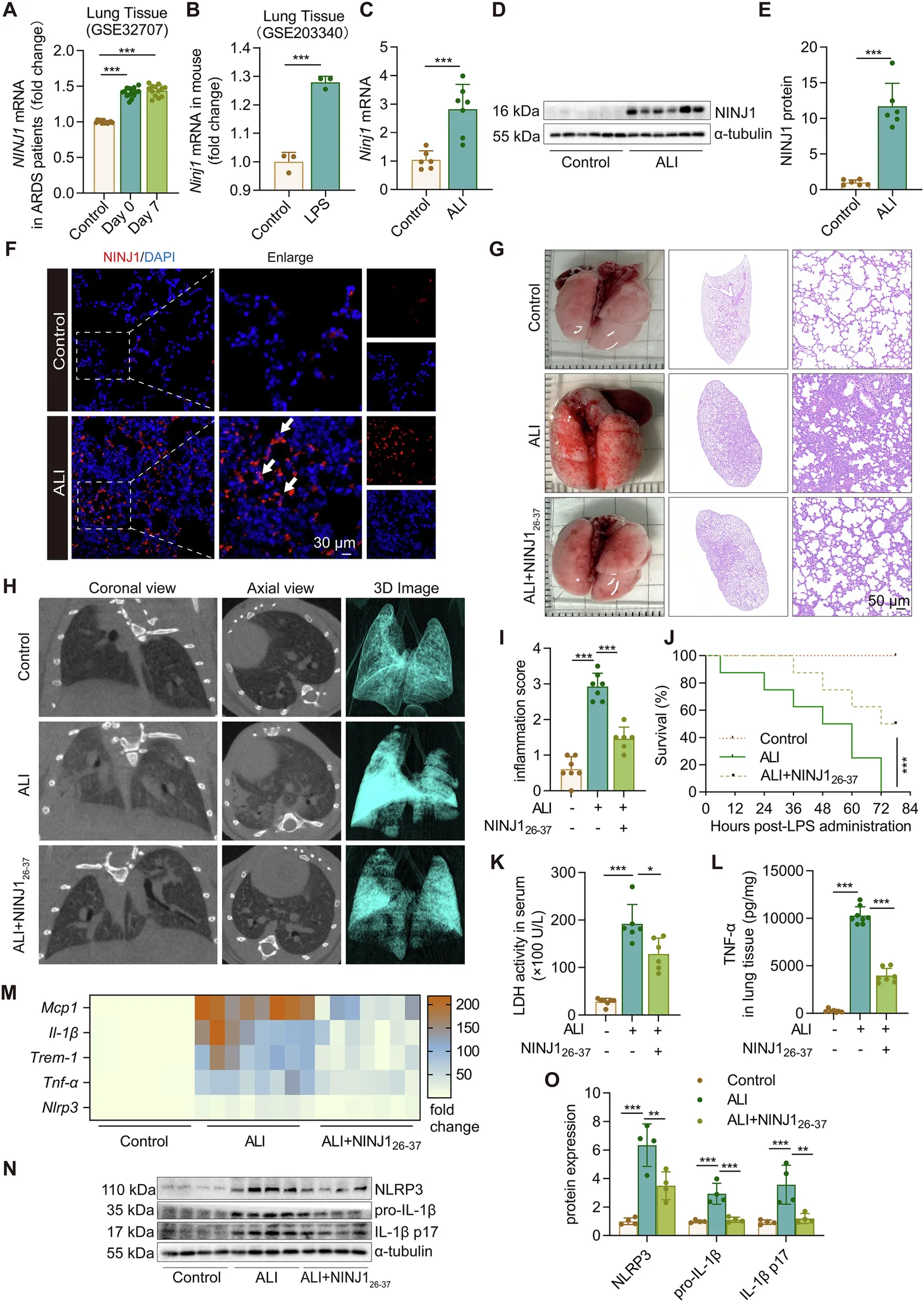
NINJ1 expression is elevated in activated neutrophils during ALI. C57BL/6J mice received an intratracheal instillation of LPS (5 mg/kg, *i.t*.). **A**–**C** Twelve hours later, mouse lungs were excised, and the mRNA and protein expression of NINJ1 were assessed by Real-time PCR and Western blot, *n* = 6. Data were shown as mean ± SD. *P < 0.05, and ***P < 0.001 (unpaired two‑tailed Student’s *t*‑test). **D** The fluorescence intensity of NINJ1 protein (red) in lungs from mice with ALI was detected by immunofluorescence, scale bar = 30 μm. **E** ALI mouse lung tissues (LPS intranasal instillation) were collected at 48 h (GSE224938). Distribution of 7 subtypes of cells by UMAP, coloured by cell types. t-SNE visualizations depict *Ninj1* expression levels, defined by binarized activity thresholds derived from AUC scores. **F** The expression of NINJ1 over all immune cells, with each point from an individual sample. **G** To elucidate the potential function of neutrophils in this ALI mouse model, a focused analysis of neutrophil populations was conducted, revealing four distinct neutrophil subsets. **H** The expression of *Ninj1* gene signatures was assessed across neutrophil subsets. **I** Neutrophil subsets trajectory analysis delineated two divergent cell destinies colored by cluster. The arrow indicated the likely course of evolution within the trajectory. **J** Monocle 2 trajectory analysis of 856 neutrophils obtained by 5′ gene expression chemistry, annotated by calculated states. **K** The heatmaps illustrated the dynamic expression patterns of genes across pseudotime, while the bar plot depicted the functional enrichment profiles of gene clusters in terminal states 5 and 6. **L** Neutrophil subpopulations in ALI lung tissues were characterized through pseudotime trajectory analysis.

Compared with patients undergoing mechanical ventilation without sepsis, SIRS, or ARDS, those diagnosed with ARDS exhibited significantly upregulated NINJ1 expression at both initial presentation (Day 0) and Day 7 of hospitalization (Fig. S1A). Consistently, elevated NINJ1 expression was observed in the LPS-induced ALI mice with lethal endotoxic shock (GSE203340) (Fig. S1B). To further investigate NINJ1 expression in the context of ALI pathogenesis, we thus induced murine ALI with LPS and evaluated its pulmonary expression. Both transcriptional and protein levels were found to be significantly upregulated (Fig. 1A–D). For single-cell profiling of NINJ1 heterogeneity and spatial distribution among immune cells during ALI, we analyzed a dataset (GSE224938) from lung tissue of ALI mice 48 h after intranasal LPS challenge. Subsequently, identification of 7 major cell subsets was achieved through the t-SNE dimension reduction method, using typical marker genes (Fig. 1E), including neutrophils (S100A8, S100A9), macrophages (CD68, CD163), dendritic cell (THBD, CD1C), B cell (CD19, CD79A, MS4A1), plasma cell (CD27, CD38), T cell (PTPRC, CD3D, CD3E), NK cells (NKG7, GNLY). Moreover, Fig. 1E right panel and Fig. 1F illustrate the *Ninj1* gene across cell subgroups using FeaturePlot analysis. Results showed that NINJ1 expression was significantly elevated in neutrophils. Consistently, single‑cell RNA‑seq analysis (GSE157789) of peripheral blood neutrophils from ARDS patients also showed high NINJ1 expression in circulating neutrophils (Fig. S2). To elucidate the functional role of NINJ1 in neutrophil biology, the neutrophil population was isolated and subdivided into four distinct subsets (Fig. 1G). NINJ1 expression was then systematically evaluated across these subpopulations. Notably, elevated NINJ1 expression was observed in all neutrophil subsets, except the Neu4 cluster (Fig. 1H). Pseudotime trajectory analysis was performed using Monocle 2 to reconstruct the temporal dynamics of neutrophil differentiation. The results indicate that the neutrophil populations originate from the Neu4 cluster, progress through the Neu3 cluster, and ultimately terminally differentiate into the Neu1 and Neu2 clusters (Fig. 1I, J). To identify key molecular regulators of neutrophil subset diversification, differential gene expression analysis was conducted on terminally differentiated populations (State 5 and State 6; Fig. 1K). Differentially expressed genes were grouped into three co-expression modules. Subsequent GO enrichment analysis demonstrated that State 6, largely composed of the Neu1 subset, was enriched in genes implicated in immune effector mechanisms. Conversely, State 5, mainly consisting of the Neu2 subset, exhibited significant enrichment of genes related to chemotaxis pathways (Fig. 1K). Based on the phenotypic characteristics of each cellular subset within the pseudotime trajectory analysis, neutrophils were further annotated into distinct functional states, including resting, active, chemotactic, and inflammatory states (Fig. 1L). NINJ1 was highly expressed in active, chemotactic, and inflammatory neutrophil subsets during ALI (Fig. 1H, L). Pulmonary cellular distribution analysis reveals that NINJ1 is enriched in neutrophils during ALI in mice.

### Hotspot identifies gene modules containing NINJ1 that are correlated with NETosis‑related genes

**Fig. 2:**
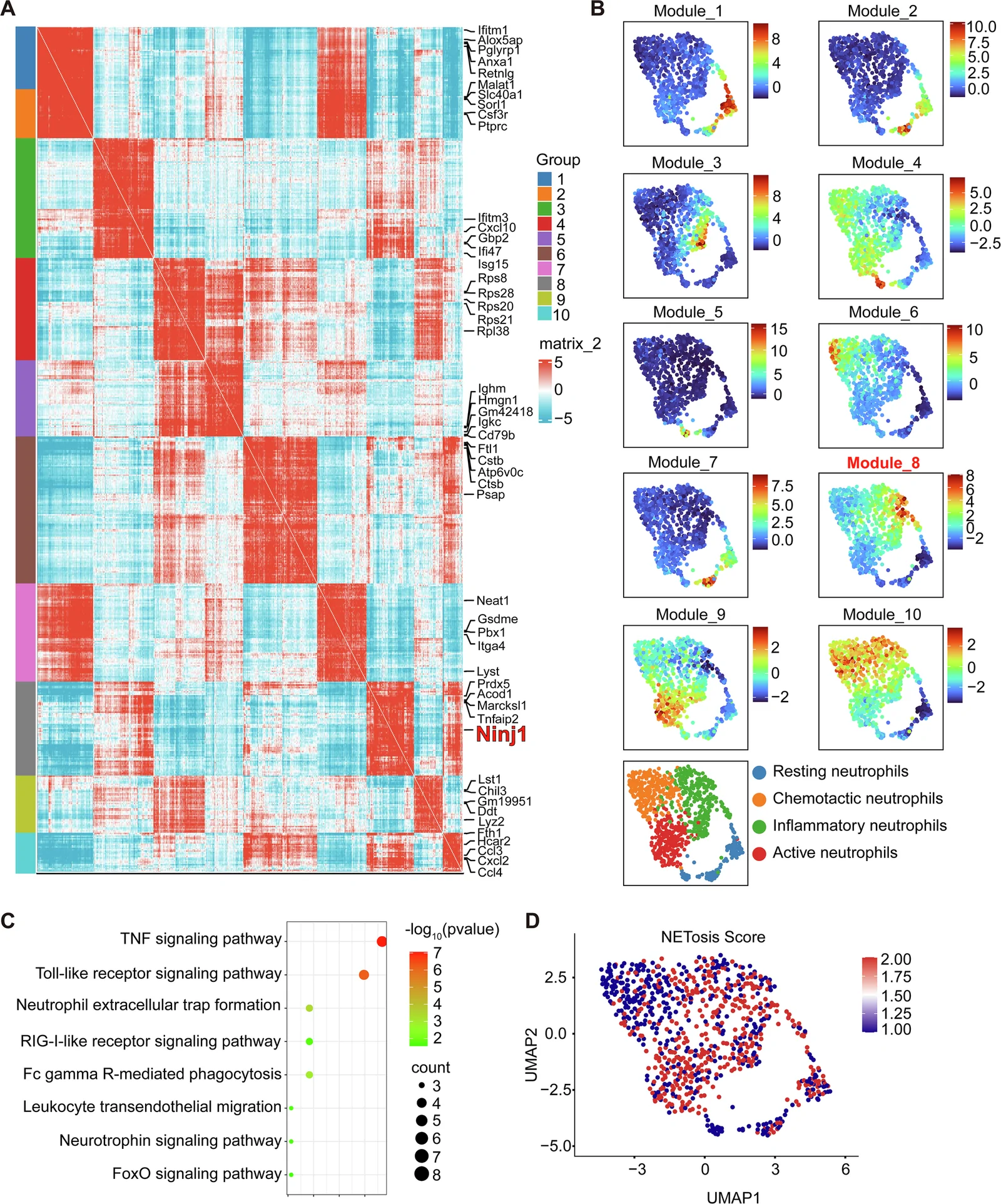
Characterization of transcriptional hotspots in neutrophils using single-cell RNA sequencing. Hotspot was used to identify spatially relevant genes within a spatial single-cell expression sample from neutrophils. **A** 1284 genes selected by Hotspot were grouped into 10 modules based on pairwise spatial correlations. **B** Gene modules were visualized with their summary, per barcode, and module scores. Beneath each module score plot, the expression level of the gene exhibiting the highest autocorrelation was shown for comparative purposes. **C** KEGG pathway enrichment analysis was performed on co-expressed genes from Module 8 to investigate neutrophil-relevant biological processes. **D** Neutrophil subpopulations were scored utilizing a NETosis-associated gene signature.

To elucidate the molecular functions and interaction networks of NINJ1 in neutrophils during ALI, we employed Hotspot analysis [35], a method designed to detect spatially restricted patterns and nonlinear covariances among genes within cellular subpopulations. This approach is particularly suitable for identifying context-specific co-expression changes within the inflammatory microenvironment of ALI. After selecting 1284 significant genes using Hotspot, we performed module identification and found that NINJ1 was uniquely assigned to module 8 (Fig. 2A; Suppl. file 1 modules_filtered_p0.001_top gene30). A total of ten gene modules were identified, with NINJ1 significantly enriched in module 8. Functional enrichment within module 8 highlighted the biological processes and regulatory pathways associated with NINJ1. Moreover, this module was upregulated explicitly in the inflammatory neutrophil subset (Fig. 2B), implying a role for NINJ1 in neutrophil inflammatory polarization during ALI. Importantly, KEGG pathway enrichment analysis of Module 8 genes revealed significant enrichment in the NETosis pathway, indicating that Module 8, which contained NINJ1, was associated with the NETosis pathway in ALI (Fig. 2C). Accordingly, a NETosis score, derived from ~40 pathway genes, was significantly elevated in both the inflammatory and active neutrophil subsets (Fig. 2D). Of note, while NINJ1 resides in module 8, it is only one component of the broader NETosis program, and its single-gene expression showed a more restricted distribution across subpopulations compared with the composite NETosis score. Together, our results identify an NINJ1-associated module that is correlated with neutrophil NETosis during ALI.

### NINJ1 oligomerization is elevated in neutrophils undergoing NETosis from ARDS patients

**Fig. 3:**
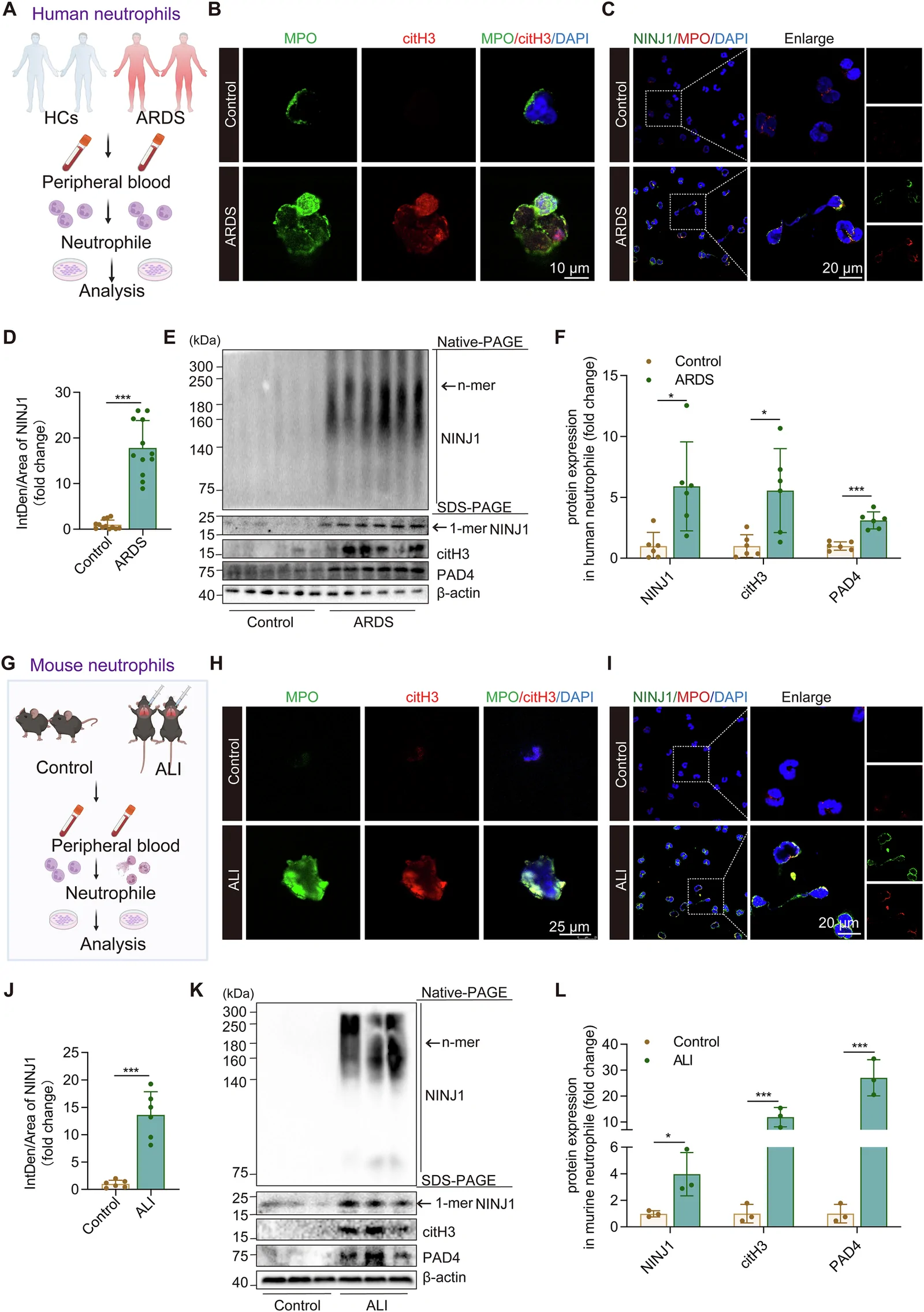
NINJ1 oligomerization was upregulated in clinical neutrophils undergoing NETosis from ARDS patients. **A** Schematic overview of experimental design for (**B**−**F**). **B** Laser-scanning confocal microscopy assessment of NETosis-related protein expression on peripheral neutrophils from HCs (*n* = 12) and ARDS patients (*n* = 12), scale bar = 10 μm. **C** IF of NINJ1 and MPO in circulating neutrophils from HCs and ARDS patients, scale bar = 20 μm. **D** Average fluorescent intensity was calculated by NINJ1^+^ fluorescence intensity (IntDen)/area of the region (Area) using ImageJ, *n* = 12. **E**, **F** Native-PAGE analysis of NINJ1 oligomerization in peripheral neutrophils from HCs and ARDS patients. SDS-PAGE analysis of NINJ1 monomer, citH3, PAD4, and β-actin was used as the internal control, *n* = 6. **G** Schematic overview of experimental design for (**H**−**L**). **H**, **I** IF images of NETosis and NINJ1 on peripheral neutrophils from non-ALI Control and ALI mice (*n* = 6). **J** Quantification of the percentage of NINJ1. **K** Native-PAGE analysis of NINJ1 oligomerization in peripheral neutrophils isolated from Control and ALI mice, *n* = 3. **K**, **L** SDS-PAGE analysis of NINJ1 monomer, citH3, PAD4, and β-actin was used as the internal control, *n* = 3. Data were shown as mean ± SD. *P < 0.05, **P < 0.01, and ***P < 0.001 (unpaired two‑tailed Student’s *t*‑test).

To further verify the results from the bioinformatics analysis, this study involved the recruitment of patients meeting the ARDS criteria alongside HCs. We detected the expression of NINJ1 in peripheral blood neutrophils from HCs and ARDS patients (Fig. 3A). Compared to neutrophils from HCs, neutrophils from ARDS patients demonstrated a marked increase in the content of NETosis’ characteristic components citH3 and MPO (Fig. 3B). We further observed a marked increase in NINJ1 fluorescence intensity co‑localized with MPO in neutrophils from ARDS patients (Fig. 3C, D). Flow cytometry analysis identified many PI-negative neutrophils from ARDS patients exhibiting high NINJ1 expression (Fig. S3). The mechanism underlying NINJ1-mediated lytic cell death involves its oligomerization, which is essential for subsequent PMR [36]. Using a biochemical native-PAGE approach that maintains native protein interactions, a band of approximately 180–300 kDa was observed in neutrophils from ARDS patients (Fig. 3E). The increase in PAD4 and citH3 suggested that cells were undergoing NETosis, and this was associated with increased total NINJ1 levels and its oligomerization (Fig. 3E, F). No alterations in pyroptosis, apoptosis, or necroptosis were detected in neutrophils from ARDS patients (Fig. S4), indicating that NINJ1 is primarily involved in NETosis rather than other lytic pathways. In the LPS-induced murine model, neutrophils were isolated from the peripheral blood of both the Control and ALI groups for IF analysis. (Fig. 3G). Neutrophils from ALI mice demonstrated a marked increase in the levels of NETosis-specific markers (citH3 and MPO), along with significantly higher NINJ1 level than those from the Control group (Fig. 3H–J). Increased NINJ1 expression was observed in neutrophils from ALI mice (Fig. 3I). Compared to Controls, both NINJ1 oligomerization and NETosis levels were significantly elevated in the peripheral blood of ALI mice (Fig. 3K, L). These results suggest that NINJ1 oligomerization is specifically correlated with NETosis during ALI/ARDS.

### *Ninj1* depletion in neutrophils alleviates LPS-induced ALI in mice

**Fig. 4:**
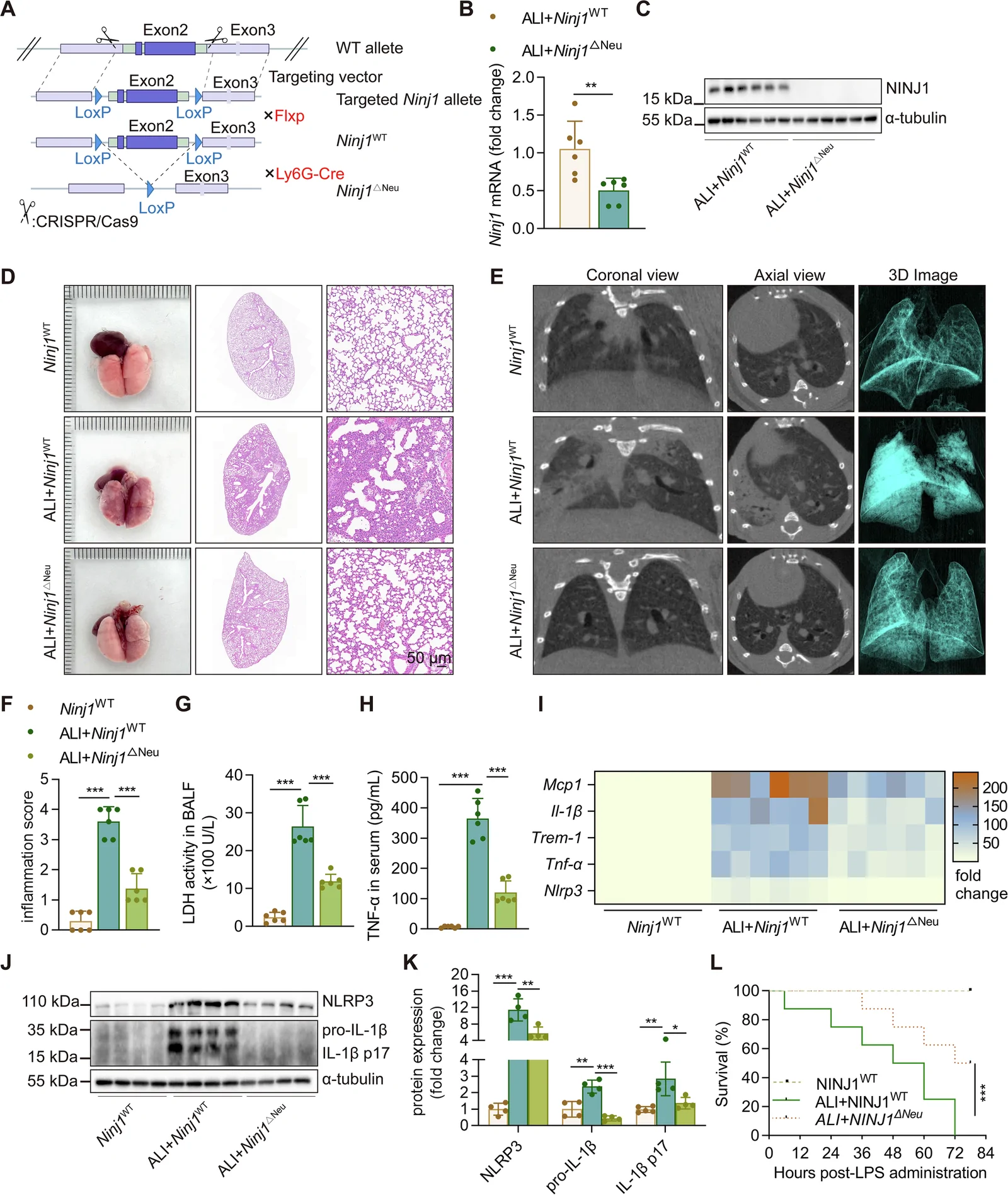
*Ninj1* depletion in neutrophils alleviated LPS-induced ALI in vivo. **A** Schematic of neutrophil-specific *Ninj1*-null mice generation. Exon 2 was floxed in the targeted allele and excised in the conditional knockout allele. *Ninj1*^WT^ (*Ly6G*-Cre-*Ninj1*^fl/fl^) and *Ninj1*^ΔNeu^ (*Ninj1*^fl/fl^/*Ly6G*^Cre^) mice were intratracheally injected with LPS and euthanized 12 h post-treatment. All assays (histology, BALF protein, TNF-α, mRNA, and protein) were performed at 12 h post‑LPS (*n* = 6). **B**, **C** Real-time PCR and Western blot were used to detect NINJ1 expression in peripheral neutrophils from *Ninj1*^WT^ and *Ninj1*^ΔNeu^ mice. **D** Representative images of the mouse lung tissue (Left panel) and the lung histopathology stained with H&E (Right panel), scale bar = 50 μm. **E** High-resolution micro-CT was utilized to assess pulmonary structural changes in a murine model of ALI. Multiplanar reconstructions, including dorsal and axial views, were obtained at the level of the T6 thoracic vertebra. Representative 3D reconstructions were generated from in vivo acquired images to visualize aerated lung regions. (*n* = 6 mice/group). **F** Inflammation score was assessed. **G** The LDH activity in the serum was detected 12 h after the LPS injection. **H** The concentration of TNF-α in lungs was detected by ELISA. **I** The mRNA expression levels of *Mcp1*, *Il-1β*, *Trem-1*, *Tnf-α*, and *Nlrp3* in lungs were quantified using Real-time PCR. **J**, **K** The protein levels of NLRP3, pro-IL-1β, and IL-1β p17 fragment in lungs from ALI mice were determined by Western blot (*n* = 6). **L** Kaplan-Meier survival curve of *Ninj1*^WT^ and *Ninj1*^ΔNeu^ following LPS challenge (25 mg/kg, *i.t*., *n* = 16 mice/group). Data were shown as mean ± SD.*P < 0.05, **P < 0.01, and ***P < 0.001 (one‑way ANOVA followed by Tukey’s post hoc test).

To investigate the function of NINJ1 on neutrophils in orchestrating ALI inflammation, neutrophil-specific *Ninj1*-deficient mice were generated by crossing *Ninj1* floxed mice with *Ly6G*-Cre mice (Figs. 4A and S5A, B). We confirmed that the NINJ1 expression was specifically deleted in neutrophils isolated from *Ninj1*^fl/fl^/*Ly6G*^Cre^ (*Ninj1*^ΔNeu^) mice, while regular expression was maintained in control *Ly6G*-Cre-*Ninj1*^fl/fl^ mice (*Ninj1*^WT^) (Fig. 4B, C). Furthermore, we verified that the NINJ1 expression was not deleted in Ly6G^-^ cells or bone marrow-derived macrophages (BMDMs) isolated from *Ninj1*^ΔNeu^ mice (Figs. S5C, S5D).

LPS-challenged *Ninj1*^ΔNeu^ mice, while not completely healthy, show markedly reduced signs of illness compared to *Ninj1*^WT^ ALI mice, including no suppurative eyes, glossier fur, and more normal posture and activity (Fig. S5E). H&E staining and micro-CT imaging confirmed that untreated *Ninj1*^ΔNeu^ mice exhibited normal lung morphology with intact alveolar structures (Fig. S6). Histological analysis revealed a marked attenuation of lung injury in *Ninj1*^ΔNeu^ mice, characterized by reduced leukocyte infiltration, alveolar congestion, and alveolar barrier thickening compared to *Ninj1*^WT^ mice after LPS challenge (Fig. 4D, F). *Ninj1*^ΔNeu^ mice exhibited a significant reduction in areas of segmental consolidation following LPS challenge (Fig. 4E). 3D reconstructions further corroborated the structural alterations, revealing markedly enlarged airspace volumes in *Ninj1*^ΔNeu^ mice compared to *Ninj1*^WT^ across all observed time points (Fig. 4E). Additionally, LDH activity in serum was reduced in *Ninj1*^ΔNeu^ mice (Fig. 4G), suggesting an attenuation of pulmonary cellular damage. The inflammatory cascade represents a hallmark characteristic of ALI. Our previous studies have identified the triggering receptor expressed on myeloid cells-1 (TREM-1) signaling pathway and NLRP3 inflammasome as a novel mechanism driving inflammatory amplification in ALI [37, 38]. The TNF-α level in serum from ALI *Ninj1*^ΔNeu^ mice was significantly lower than that of *Ninj1*^WT^ mice (Fig. 4H). *Ninj1*^ΔNeu^ mice exhibited significant downregulation of key inflammatory mediators associated with ALI, including *Mcp1*, *Il-1β*, *Trem-1*, *Tnf-α*, and *Nlrp3* mRNA (Fig. 4I). The protein levels of NLRP3, pro-IL-1β, and IL-1β p17 in the lungs were significantly decreased in *Ninj1*^ΔNeu^ mice (Fig. 4J, K), reflecting the overall inflammatory status of the lung tissue. *Ninj1*^ΔNeu^ mice exhibited a significantly increased long-term survival rate following ALI induction (Fig. 4L). These results suggest that NINJ1 in neutrophils is a key factor in the pathophysiology of organ dysfunction associated with ALI.

### NINJ1 drives NET release in neutrophils during ALI

**Fig. 5:**
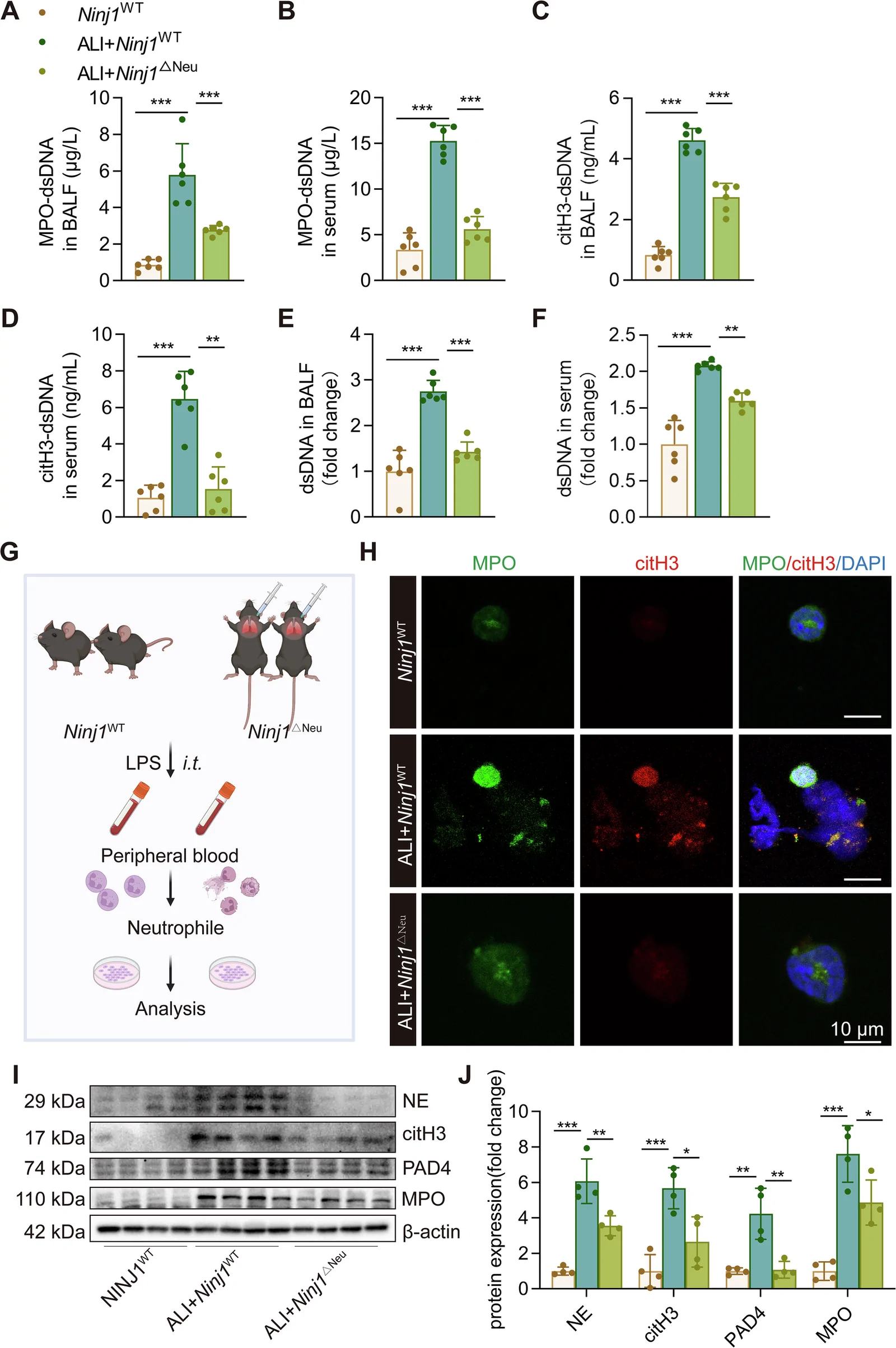
Neutrophil-specific *Ninj1* depletion inhibits NET release during ALI. *Ninj1*^WT^ and *Ninj1*^ΔNeu^ mice were administered with LPS for 12 h (5 mg/kg, *i.t*.). **A**–**D** The levels of NETs components MPO-dsDNA and citH3-dsDNA in BALF and serum detected by ELISA. **E**, **F** dsDNA in BALF and serum was measured using the PicoGreen® dsDNA Quantification Kit. **G** Schematic overview of experimental design for **H**–**J**. **H** Laser-scanning confocal microscopy assessment of NETosis-related protein expression on peripheral neutrophils from ALI mice, scale bar = 10 μm. **I**, **J** Western blot was used to detect NE, citH3, PAD4, and MPO expression in neutrophils isolated from *Ninj1*^WT^ and *Ninj1*^ΔNeu^ mice, *n* = 6 mice/group. Data were shown as mean ± SD. *P < 0.05, **P < 0.01, and ***P < 0.001 (one‑way ANOVA followed by Tukey’s post hoc test).

We further investigated the role of NINJ1 in NET release during ALI in vivo. BALF reflects the soluble components within the alveolar space, including molecules released from cells. ELISA analysis showed that after LPS challenge, the levels of MPO-dsDNA and citH3-DNA complexes, as well as dsDNA, in both BALF and serum were significantly lower in *Ninj1*^ΔNeu^ mice than in *Ninj1*^WT^ Control (Fig. 5A–F). Consistently, a significant reduction in MPO and citH3 double‑positive NETs, as well as reduced MPO activity, was observed in the lung tissue of *Ninj1*^ΔNeu^ mice (Fig. S7), indicating reduced neutrophil activation. In the complex ALI lung microenvironment, cytokines, chemokines, and DAMPs regulate neutrophil recruitment. NETs, as DAMPs, amplify inflammation *via* a positive feedback loop. Peripheral blood neutrophils were isolated from *Ninj1*^WT^ and *Ninj1*^ΔNeu^ mice (Fig. 5G). We found that the fluorescence intensity of MPO and citH3 in *Ninj1*^ΔNeu^ mice was significantly diminished compared to that in *Ninj1*^WT^ mice after ALI induction (Fig. 5H). Western blot analysis revealed a significant decrease in the levels of NETs protein, such as NE, citH3, PAD4, and MPO in neutrophils from *Ninj1*^ΔNeu^ mice compared with *Ninj1*^WT^ mice (Fig. 5I, J). These results demonstrate that neutrophil-specific *Ninj1* depletion prevents NET release and pulmonary dysfunction, improving ALI outcomes.

### NINJ1 is indispensable for NETosis-related PMR

**Fig. 6:**
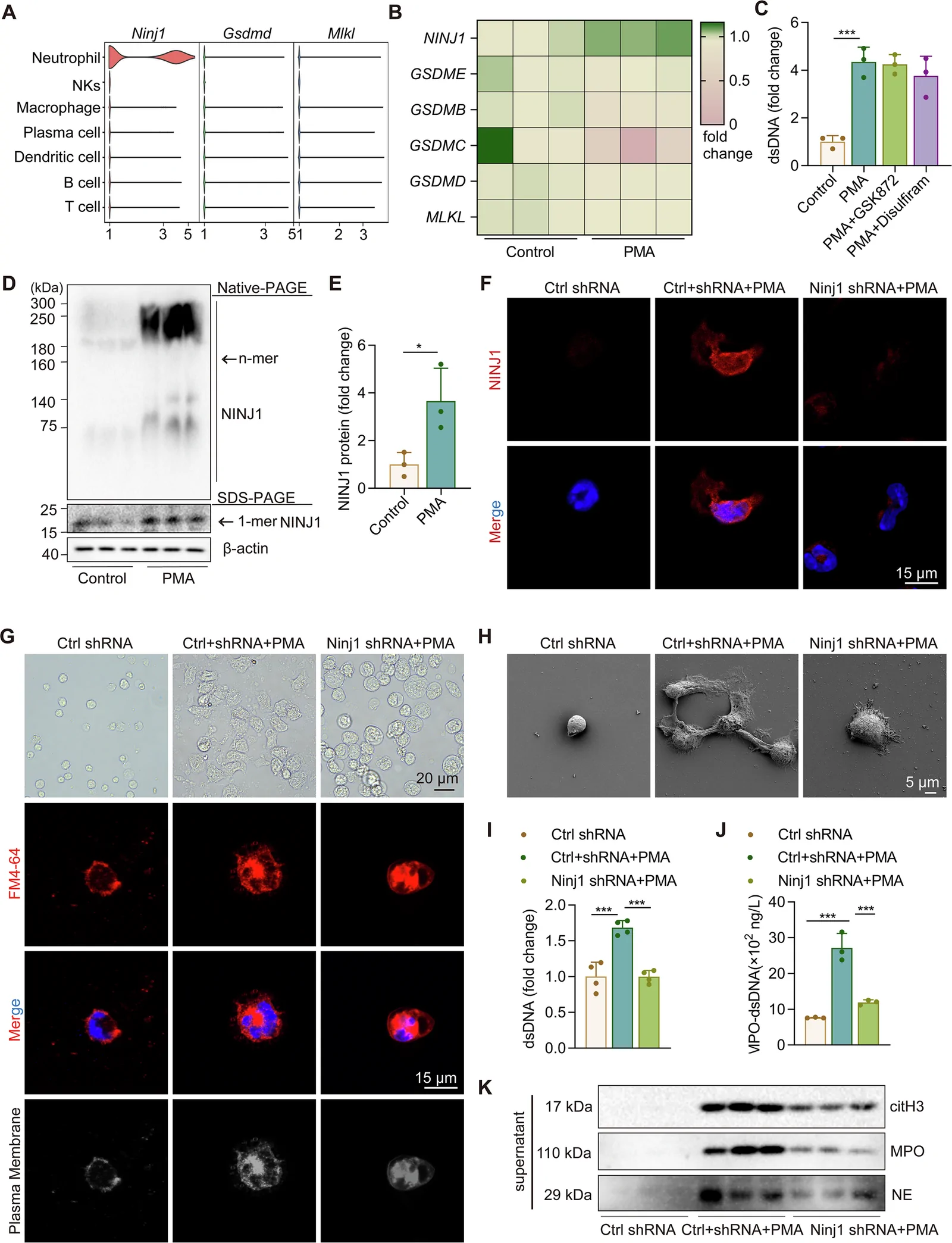
NINJ1 deficiency prevents PMR and NET release *in vitro.* **A** The expression of pore-forming, including *Ninj1*, *Gsdmd*, and *Mlkl*, in overall immune cells (GSE224938). **B** Neutrophils isolated from human donors were stimulated with PMA (50 nM) for 3 h and subsequently harvested for transcriptomic sequencing analysis. RNA-seq analyses revealed the expression of pore-forming proteins, including *NINJ1*, *GSDME*, *GSDMB*, *GSDMC*, *GSDMD*, and *MLKL*, in neutrophils undergoing PMA-induced NETosis. **C** Mouse primary neutrophils were treated with GSK872 (an MLKL inhibitor, 1 μM), or Disulfiram (a GSDMD inhibitor, 5 μM) 30 min before PMA (50 nM, 3 h) induction, and the levels of dsDNA were quantified, *n* = 3. Data were shown as mean ± SD. ***P < 0.001 (one‑way ANOVA followed by Tukey’s post hoc test). **D** Unless otherwise stated, dHL‑60 cells were treated with 100 nM PMA for 3 h. Native-PAGE analysis of NINJ1 oligomerization in PMA-treated dHL-60 cells. SDS-PAGE analysis of NINJ1 monomer. **E** Quantification of NINJ1 monomer protein levels, *n* = 3. Data were shown as mean ± SD. *P < 0.05 (unpaired two‑tailed Student’s *t*‑test). **F** Immunofluorescence images of NINJ1 on PMA-treated dHL-60 cells, scale bar = 15 μm. **G** The corresponding brightfield image was shown in live cells labeled with the plasma membrane dye FM4-64. Assessment of plasma membrane integrity by laser-scanning confocal microscopy in neutrophils transfected with either Ctrl shRNA or *Ninj1* shRNA after the PMA stimulation, scale bar = 15 μm or 20 μm. **H** Representative scanning electron microscopy images of neutrophil morphology in dHL-60 cells. **I** dsDNA in supernatant was measured using the PicoGreen® dsDNA Quantification Kit. **J** The concentrations of MPO-dsDNA in the neutrophil culture supernatant were determined using an ELISA test. **K** The levels of citH3, MPO, and NE in the supernatant were detected by Western blot. *n* = 3, Data were shown as mean ± SD. *P < 0.05 and ***P < 0.001 (one‑way ANOVA followed by Tukey’s post hoc test).

To identify and characterize the specific pore-forming proteins during NETosis-related PMR, we analyzed an available single-cell RNA sequencing dataset (GSE224938). This analysis revealed that *Ninj1* expression was markedly higher than that of other pore-forming proteins, including *Gsdmd* and *Mlkl*, in mouse neutrophils (Fig. 6A). To further verify the conclusions from the single-cell RNA sequencing dataset, primary human neutrophils were isolated and stimulated with PMA for RNA sequencing analysis. This analysis revealed the differential expression of pore-forming proteins, including *NINJ1*, *GSDME*, *GSDMB*, *GSDMC*, *GSDMD*, and *MLKL*, in PMA-treated neutrophils. Notably, only NINJ1 was significantly upregulated during PMA-triggered NETosis (Fig. 6B). Consistently, real‑time PCR analysis of LPS‑primed mouse neutrophils showed that NINJ1 was the only pore‑forming protein gene upregulated (Fig. S9). Previous studies indicated that NINJ1 overexpression is required to trigger spontaneous cell lysis [20], suggesting that the upregulated NINJ1 expression in neutrophils serves as the prerequisite for NINJ1 oligomerization and subsequent PMR. Subsequent pharmacological inhibition of MLKL using GSK872, or of GSDMD using disulfiram, failed to suppress extracellular dsDNA release in PMA-treated neutrophils (Fig. 6C). In PMA‑stimulated mouse primary neutrophils, the upregulation of citH3 and NINJ1 from 2 hours without caspase 3 alteration (Fig. S10) definitively demonstrates that these cells undergo programmed suicidal NETosis, a process distinct from secondary necrosis. We also observed upregulation and oligomerization of NINJ1 in PMA-treated dHL-60 cells (Fig. 6D–F) and primary neutrophils (Fig. S11A, B), as well as in LPS‑stimulated mouse primary neutrophils (Fig. S13A). Morphologically, PMA-treated neutrophils with NINJ1 shRNA exhibited a persistent ‘bubble’ morphology (Fig. 6G, upper panel). PMA-treated NINJ1-silenced neutrophils developed an intact plasma membrane ballooning morphology (Fig. 6G, lower panel). Scanning electron microscopy revealed that, in the PMA + NINJ1 shRNA group, neutrophils exhibited reduced membrane extension and fewer extracellular net‑like structures compared to PMA treatment alone. However, they still displayed more pronounced pseudopodia than the control shRNA group (Fig. 6H). NINJ1 shRNA administration reduced dsDNA and MPO-dsDNA complex levels in the culture supernatants of dHL-60 treated with PMA (Fig. 6I, J). Immunofluorescence analysis further revealed that NINJ1 silencing markedly reduced the extracellular co‑localization of citH3, MPO, and DNA in both PMA‑treated (Fig. S12A) and LPS‑treated neutrophils (Fig. S13B), indicating decreased NET release. Notably, the components were measured in the cell culture supernatant to reflect the NET release. A significant reduction in the supernatant levels of citH3, MPO, and NE was observed in NINJ1-silenced neutrophils (Fig. 6K). Furthermore, analysis of total cell lysates showed that NETosis‑associated protein expression (citH3, MPO, and NE) was not altered in NINJ1-silenced neutrophil (Fig. S15), indicating that NINJ1 predominantly acts through pore formation to mediate NET release. Together, our data support that neutrophil PMR and subsequent NET release during NETosis occur in a NINJ1-dependent manner.

### K45 and N60 are critical for NINJ1 oligomerization and subsequent NET release

**Fig. 7:**
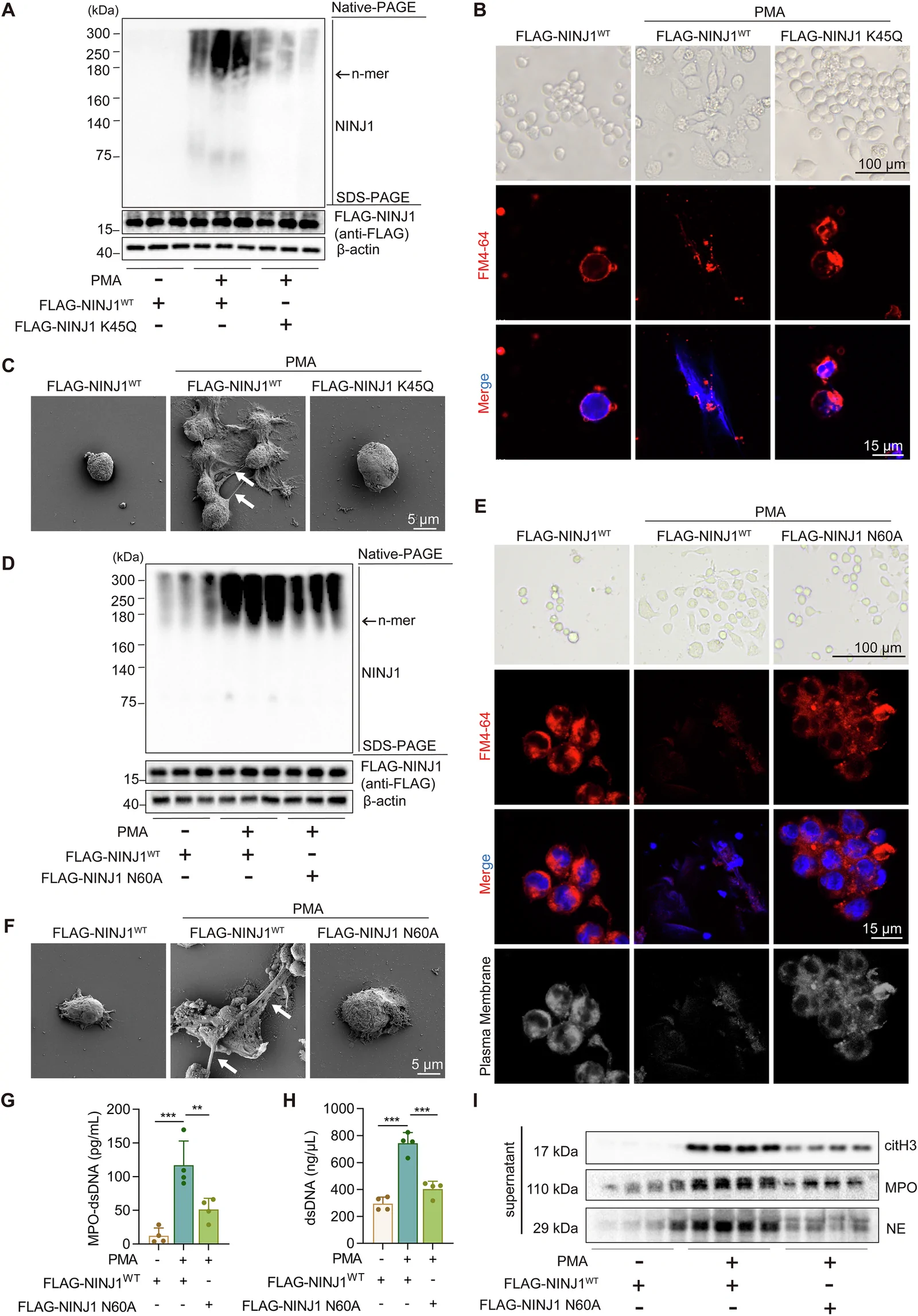
K45 and N60 are required for NINJ1 oligomerization and NET release. Lysine (K) residues were mutated to glutamine (Q). **A** Native-PAGE was employed to assess NINJ1 oligomerization in neutrophils individually transfected with either NINJ1^WT^ or the NINJ1-K45Q mutant. **B** Corresponding brightfield image of live cells stained with the plasma membrane dye FM4-64. Plasma membrane integrity was further evaluated by laser-scanning confocal microscopy in neutrophils expressing either NINJ1^WT^ or the NINJ1-K45Q mutant after PMA stimulation, scale bar = 15 μm. **C** Scanning electron microscopy images depict the morphological features of neutrophils expressing either NINJ1^WT^ or the NINJ1-K45Q mutant with PMA stimulation. **D** Asparagine sites (N) were mutated to alanine (**A**). NINJ1 oligomerization is impaired by the N60A mutation (Native‑PAGE). **E** Brightfield and FM4‑64 staining of NINJ1‑N60A neutrophils after PMA stimulation, scale bar = 15 μm. **F** SEM images of PMA‑stimulated NINJ1‑N60A neutrophils. **G** The concentrations of MPO-dsDNA complexes in culture supernatants after PMA treatment were measured by ELISA. **H** Extracellular dsDNA release was quantified using the PicoGreen® dsDNA assay. **I** The presence of citH3, MPO, and NE in supernatants was confirmed by Western blot analysis. *n* = 3–4, Data were shown as mean ± SD. **P < 0.01 and ***P < 0.001 (one‑way ANOVA followed by Tukey’s post hoc test).

To validate the functional importance of NINJ1 oligomerization in NET release, we generated a K45Q mutation, which targets a residue located at the core interface of the activated NINJ1 oligomer based on the cryo‑EM structure (3.0 Å resolution) [20, 22, 39]. Native‑PAGE analysis revealed that the K45Q mutation specifically blocked the formation of PMA‑induced NINJ1 oligomers (Fig. 7A). Consistent with the observation in Ninj1-silencing neutrophils, neutrophils with NINJ1-K45Q mutant sustained the ballooned morphology (Fig. 7B). Scanning electron microscopy further showed abundant extracellular net‑like structures surrounding PMA‑stimulated NINJ1^WT^ neutrophils, which were markedly diminished in both NINJ1‑K45Q mutant cells (Fig. 7C). PMA‑stimulated dHL‑60 cells expressing NINJ1^WT^ showed extensive NET release, as evidenced by the presence of extracellular DNA decorated with citH3 and MPO, whereas the NINJ1‑K45Q mutant exhibited markedly reduced NET release (Fig. S14). N60 is simultaneously involved in maintaining the resting‑state autoinhibitory dimer and stabilizing the activated‑state oligomer through an interaction network with S87, Q91, and N133 [40]. Consistently, the N60A mutation impaired NINJ1 oligomerization (Fig. 7D), sustained the ballooned morphology (Fig. 7E), and markedly reduced extracellular net‑like structures (Fig. 7F) as well as supernatant levels of MPO‑dsDNA, dsDNA, citH3, MPO, and NE upon PMA stimulation (Fig. 7G–I). The NINJ1‑N60A mutant similarly reduced NET release in PMA‑stimulated dHL‑60 cells (Fig. S16B). In addition, analysis of total cell lysates revealed that the expression of citH3, MPO, and NE, involved in NETosis‑related proteins, was not altered by the N60A mutation (Fig. S15). These findings highlight NINJ1 oligomerization as a critical step for NET extrusion and establish K45 and N60 as key residues governing NINJ1 assembly and function in neutrophils.

## Discussion

This study demonstrates that the pore-forming protein NINJ1 mediates NETs extrusion and contributes to pulmonary dysfunction in ALI. Analysis of a public single-cell RNA sequencing dataset indicated that NINJ1 is highly expressed primarily in neutrophils during ALI. Hotspot analysis further connected NINJ1 to a functional module related to NETosis. In LPS-induced ALI models, either genetic deletion of *Ninj1* in neutrophils reduced NET release, alleviated lung injury, and decreased the extent of segmental consolidation and airspace pathology. Notably, neutrophils from ARDS patients undergoing NETosis showed NINJ1 oligomerization on the plasma membrane and within canonical NET structures. Mechanistic studies revealed that K45 and N60 are critical for NINJ1 oligomerization and subsequent NET release.

Extracellular NETs have been identified as a DAMP in ARDS/ALI, but the release mechanism remains unclear. We have identified NINJ1 as a key pore-forming protein for NET release. Previous studies suggested that caspase-11/GSDMD mediates NET release [41]. PMR following GSDMD pore formation was regarded as a passive consequence of osmotic imbalance [42]. However, the discovery of NINJ1 has shifted this paradigm, revealing PMR as an actively executed process [23]. Our results demonstrated that NETs’ extrusion during ALI is a NINJ1-dependent event. Consistent with this, comparative immunophenotyping and single-cell transcriptomic analyses have identified NINJ1 as a candidate biomarker of severity in both influenza- and COVID-19-induced lung injury [43]. Functionally, NINJ1 promotes PMR in response to mechanical stress, which may originate from GSDMD-induced osmotic imbalance or lipid peroxidation [44]. Thus, we propose that plasma membrane rupture requires the coordinated action of both small and large pore-forming proteins, in which GSDMD-formed small pores alter membrane tension, thereby triggering NINJ1 activation. Furthermore, This view is consistent with prior studies. Xie et al. [45] and Chen et al. [46] showed GSDMD involvement in NETosis, while Schachter et al. [47] revealed that GSDMD mediates early events (ATP release) and NINJ1 executes late-stage PMR. Integrating these findings, we propose a temporal model: GSDMD primarily contributes to early steps of NETosis, including nuclear membrane permeabilization, chromatin decondensation, and partial content release, whereas NINJ1 acts downstream as the final effector of plasma membrane rupture, enabling complete NET release. Importantly, regardless of the upstream trigger (PMA or LPS), NINJ1-mediated membrane rupture represents a convergent terminal step required for full NET release.

Previous studies have reported that inhibiting NINJ1 or *Ninj1* deficiency reduces tissue injury and neutrophil infiltration. For instance, NINJ1 inhibition limits liver ischemia‑reperfusion injury by reducing neutrophil recruitment [48]. Myeloid‑specific *Ninj1* deletion similarly reduces neutrophil infiltration, where NINJ1 is proposed to act as a membrane protein regulating Kupffer cell responses and CXCL1 production [49]. In traumatic brain injury, NINJ1 knockdown mitigates neutrophil infiltration and NETosis formation, but the study relies on tissue‑level immunofluorescence without directly demonstrating a role for NINJ1 in PMR‑mediated NET release [29]. In oxalate nephropathy, myeloid-specific *Ninj1* deletion reduces neutrophil infiltration and NETosis-associated gene expression [50]. Thus, while NINJ1 has been implicated in neutrophil recruitment or activation at the tissue level, direct evidence linking NINJ1‑mediated PMR to NET release has been lacking. In contrast, our study identified that neutrophils are a cell type expressing high levels of NINJ1 during ALI. Using neutrophil‑specific *Ninj1* knockout mice, we showed that targeted NINJ1 deletion alleviates lung injury. More importantly, we directly demonstrated that NINJ1‑mediated PMR is required for NET extrusion through plasma membrane staining, electron microscopy, and quantification of supernatant NET components. To contextualize this finding, it is important to consider the roles of other pore-forming proteins. GSDMD serves as the executor of pyroptosis [49], while MLKL executes necroptosis [51]. NINJ1 has recently been identified as a key mediator of lytic cell death, and its membrane‑permeabilizing function is independent of the pore‑forming molecules MLKL, GSDMD, and GSDME [52]. In certain NETosis pathways, such as those triggered by intracellular LPS, GSDMD acts upstream by facilitating nuclear envelope permeabilization and chromatin decondensation. However, NETs are large macromolecular complexes, and their expulsion requires a catastrophic rupture of the plasma membrane. NINJ1 functions as the terminal physical mediator of this membrane rupture, an essential step for releasing large DNA‑protein complexes into the extracellular space. Notably, MLKL does not participate in NET release [17]. Our study shows that during NETosis, NINJ1 is the most highly expressed among these three pore‑forming proteins, and its silencing, but not inhibition of GSDMD or MLKL, dramatically suppresses NET release. Future studies are required to dissect whether NINJ1 modulates neutrophil activation, chemotaxis, or participates in endothelial adhesion and vascular permeability in ALI.

The precise mechanisms triggering NINJ1 oligomerization, which is crucial for PMR in lytic cell death, remain elusive. Palmitoylation-mediated activation of GSDMD is critical for its membrane localization and pore formation during pyroptosis [53]. MLKL causes necrotic membrane disruption upon phosphorylation by RIP3 [51]. By analogy, NINJ1 may also undergo post-translational modification during its activation. Based on the structural features of NINJ1 documented in UniProt, CSSPalm-based bioinformatic analysis predicted a lack of acetylation and palmitoylation sites, but indicated potential phosphorylation at Thr18, Ser25, Ser46, and Ser50. However, our phosphoproteomic profiling of PMA-treated neutrophils identified 2303 upregulated phosphoproteins, none corresponding to NINJ1, as shown in Fig. S17. To directly assess the role of NINJ1 oligomerization in NET release, we generated a K45Q mutation targeting a residue located at the core interface of the activated NINJ1 oligomer, based on the cryo‑EM structure (3.0 Å resolution) [20, 22]. This mutation specifically blocked PMA‑induced NINJ1 oligomerization and significantly reduced NET release. These results indicate that K45 is essential for the assembly of functional NINJ1 oligomers during NET release. We also investigated N60, which was previously reported to be an N‑glycosylation site implicated in NINJ1 homodimer formation [54]. More recent structural studies have refined our understanding of this residue. Our results indicate that NINJ1 is highly conserved between mouse and human (89.5% identity, 95.4% homology), and that the N60 N‑glycosylation motif is conserved across rodents, humans, and other vertebrates, as summarized in Suppl. Table 2. The originally described homodimer is now recognized as the autoinhibited form of NINJ1 [39], and topological predictions place N60 in the intracellular region, raising questions about whether it can be glycosylated at all. Furthermore, N60 plays a direct structural role in the activated oligomeric state by participating in an interaction network with a conserved triad of residues (Ser87, Gln91, and Asn133) that stabilizes the oligomer [40]. Our mutational analysis showed that the N60A mutation impaired NINJ1 oligomerization and markedly reduced NET release. Whether N60 functions *via* N‑glycosylation or through direct structural stabilization remains to be determined. Nevertheless, both K45 and N60 are critical for NINJ1 oligomerization and subsequent NET release.

Several limitations should be considered. First, the precise triggers of NINJ1 oligomerization and its mechanism of mediating PMR remain unknown. NINJ1 oligomerization occurs across various cell death pathways, suggesting a conserved upstream signal [39]. In multiple lytic inflammatory cell death pathways, such as pyroptosis and ferroptosis, calcium (Ca²⁺) influx serves as a prerequisite event that is both necessary and antecedent to NINJ1 oligomerization and subsequent PMR [52, 55]. Ca^2+^ signaling also represents a principal trigger for NETosis [56]. Whether calcium initiates NINJ1‑mediated NET release warrants future investigation. Furthermore, while the present study focused primarily on neutrophils, the role of NINJ1 in lung parenchymal cells, such as alveolar epithelial cells, during ALI remains to be investigated, representing an important direction for future studies. Finally, the two postoperative ARDS samples may still be confounded by surgical trauma, anesthesia, and transfusion, and the small cohort size (*n* = 12) limits our findings to preliminary observations of NINJ1 trends, which await validation in larger, more diverse ARDS populations.

## Conclusion

Collectively, our study demonstrated that the pore-forming protein NINJ1 is active in neutrophils from ALI mice and humans and plays a crucial role in NET release and organ dysfunction during ARDS/ALI.

## Supplementary information

Original Images for Blots

Supplementary file 1 modules_filtered_p0.001_top gene30. csv

Supplementary materials.docx

A reproducibility checklist

Supplementary File 2-Clinical Data.xlsx

## Data Availability

The datasets supporting the conclusions of this article are included within the article and its additional file. For any further data requests, please get in touch with the corresponding author.
