# Supplementary material for: NINJ1 plays a vital role in the release of neutrophil extracellular traps during acute lung injury
Source: Cell Death Dis. 2026 Jun 25;17(1):787. doi: 10.1038/s41419-026-08995-5 (PMC13558649; doi:10.1038/s41419-026-08995-5)

Figure 1

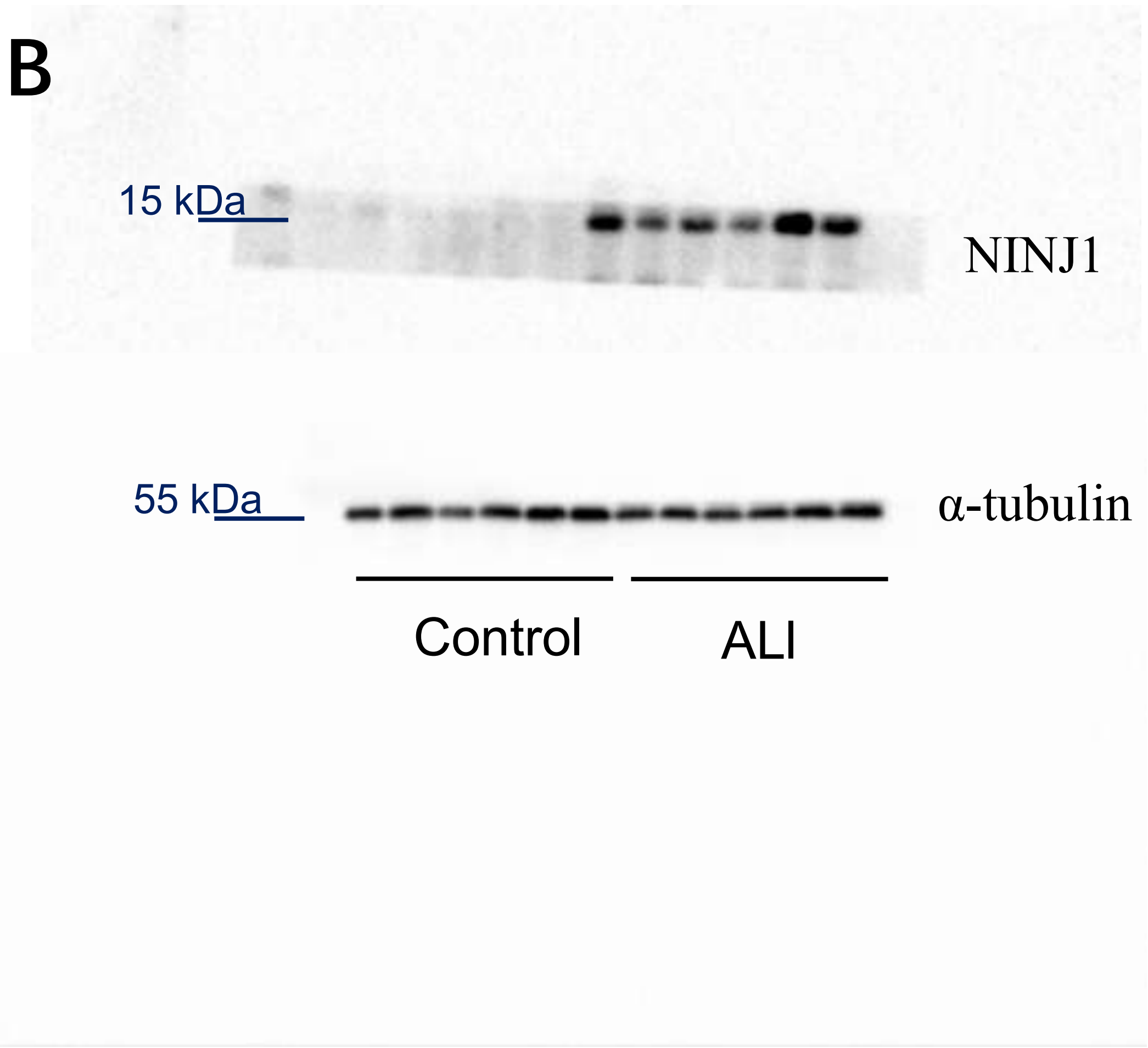

Figure 3

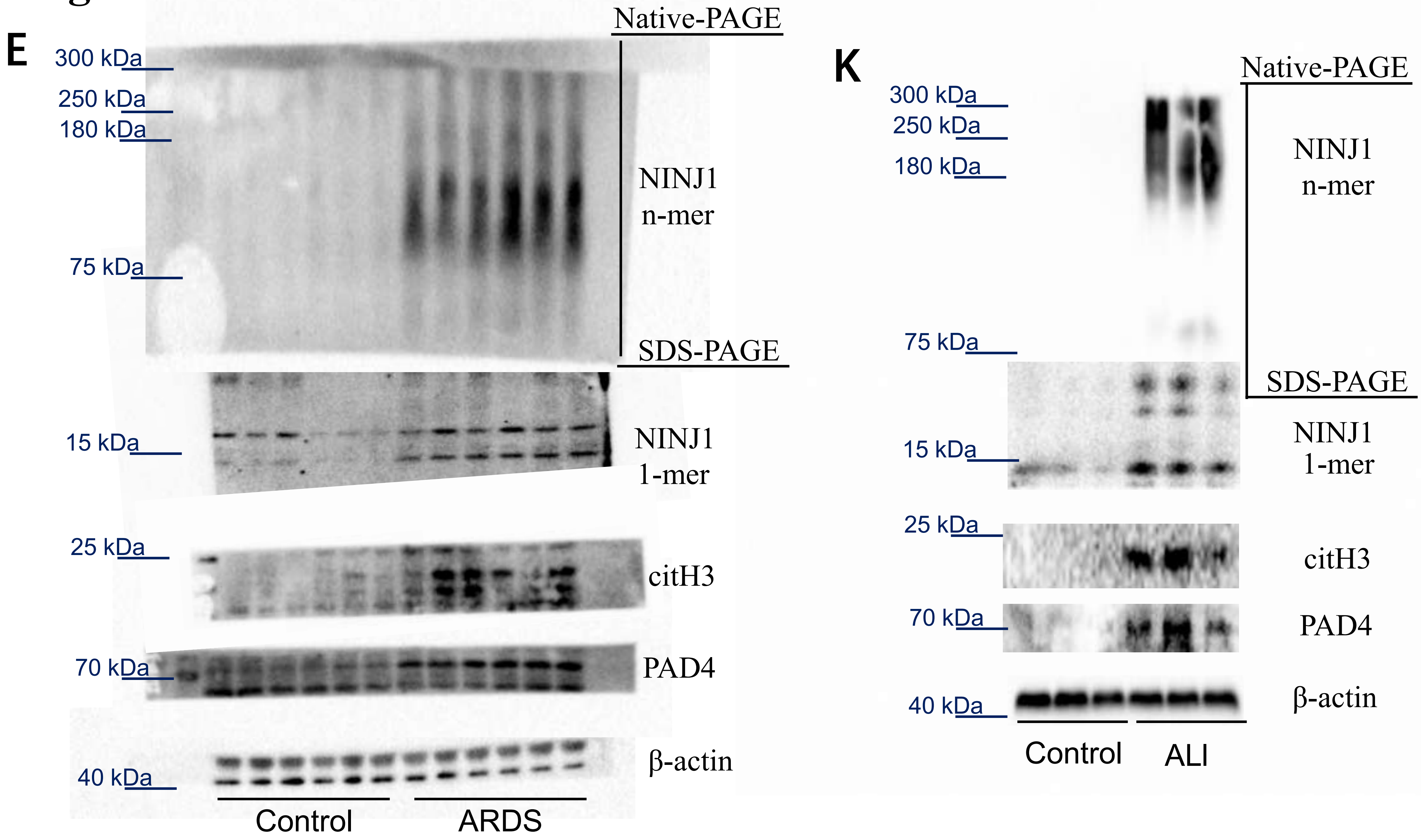
E
300 kDa
250 kDa
180 kDa
75 kDa
Native-PAGE
NINJ1
n-mer
SDS-PAGE
15 kDa
NINJ1
1-mer
25 kDa
citH3
70 kDa
PAD4
40 kDa
β-actin
Control
ARDS
K
Native-PAGE
300 kDa
250 kDa
180 kDa
NINJ1
n-mer
75 kDa
SDS-PAGE
15 kDa
NINJ1
1-mer
25 kDa
citH3
70 kDa
PAD4
40 kDa
β-actin
Control
ALI

Figure 4

C

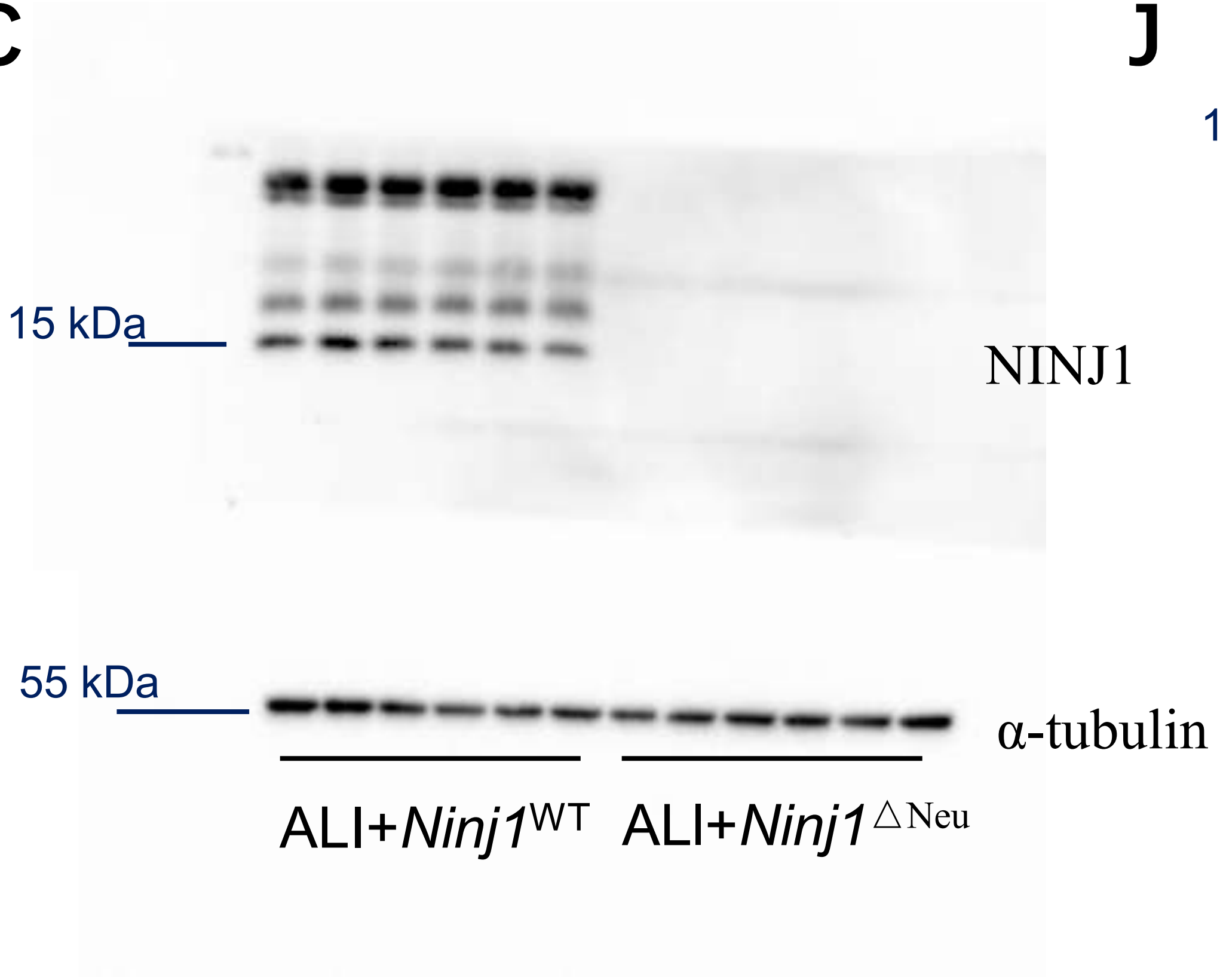
15 kDa
NINJ1
55 kDa
α-tubulin
ALI+Ninj1WT
ALI+Ninj1△Neu

J

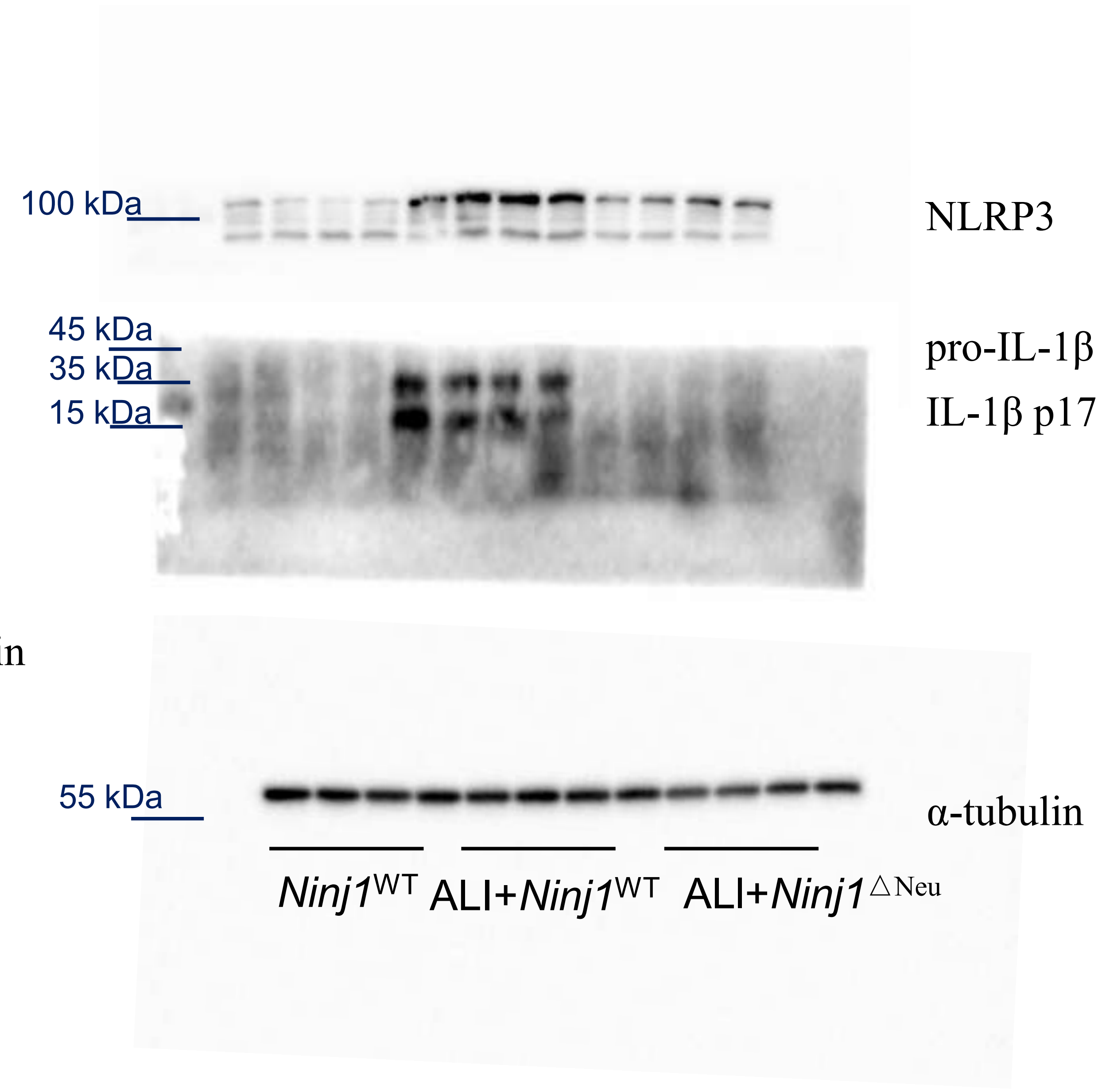
100 kDa
NLRP3
45 kDa
35 kDa
15 kDa
pro-IL-1β
IL-1β p17
55 kDa
α-tubulin
Ninj1WT
ALI+Ninj1WT
ALI+Ninj1△Neu

Figure 5

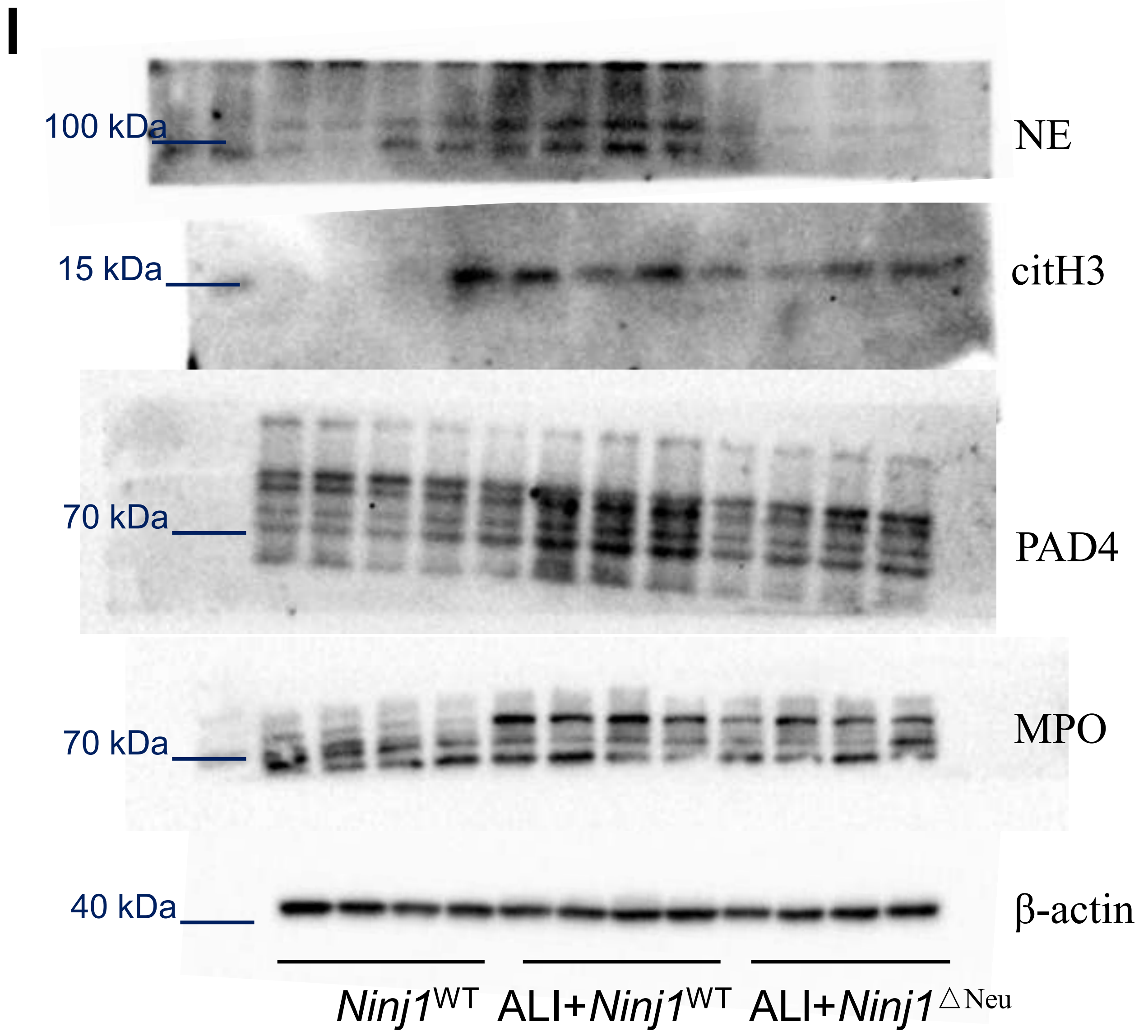

I
100 kDa
NE
15 kDa
citH3
70 kDa
PAD4
70 kDa
MPO
40 kDa
β-actin
Ninj1WT
ALI+Ninj1WT
ALI+Ninj1△Neu

Figure 6

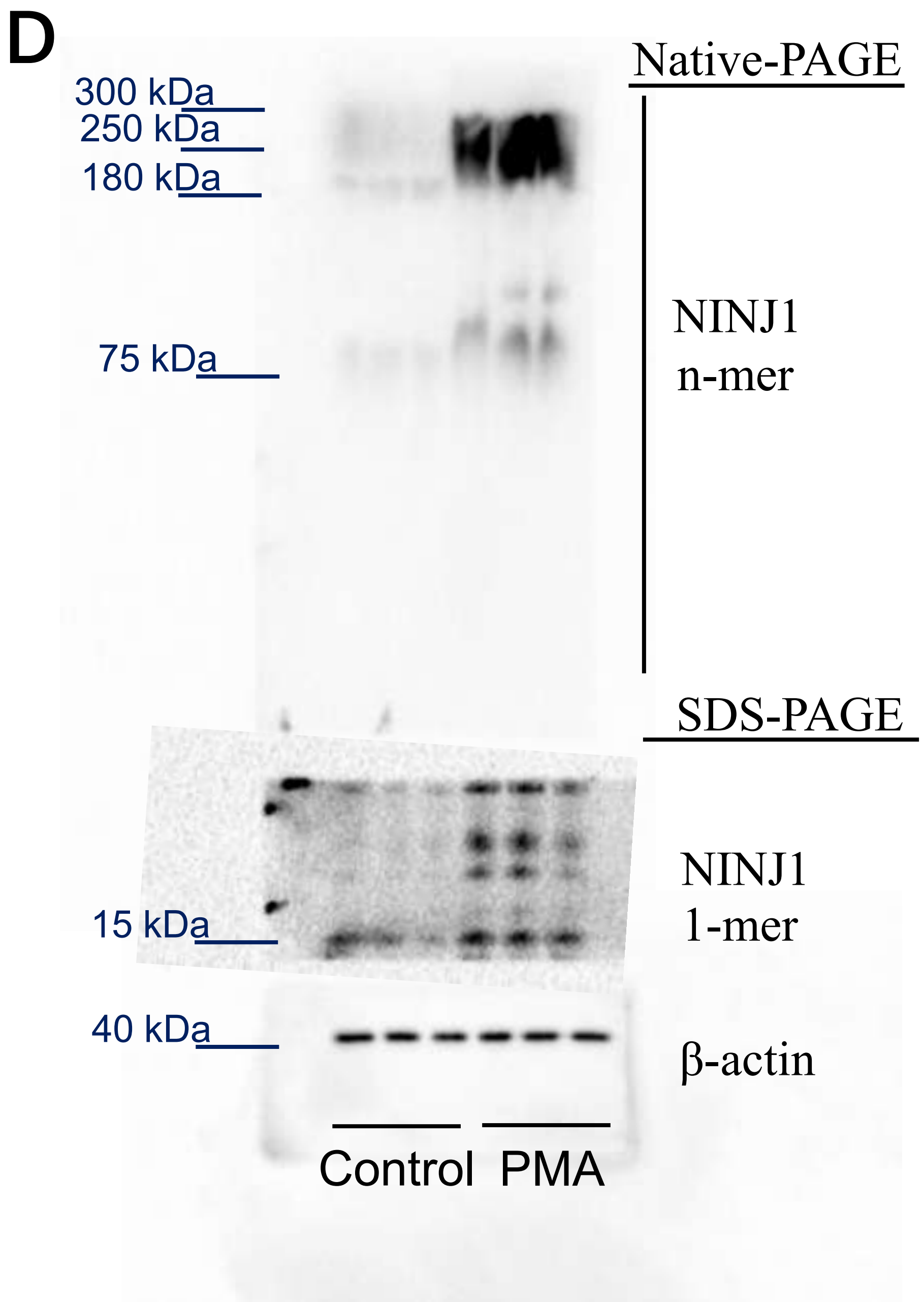
D
300 kDa
250 kDa
180 kDa
75 kDa
15 kDa
40 kDa
Native-PAGE
NINJ1
n-mer
SDS-PAGE
NINJ1
1-mer
β-actin
Control
PMA

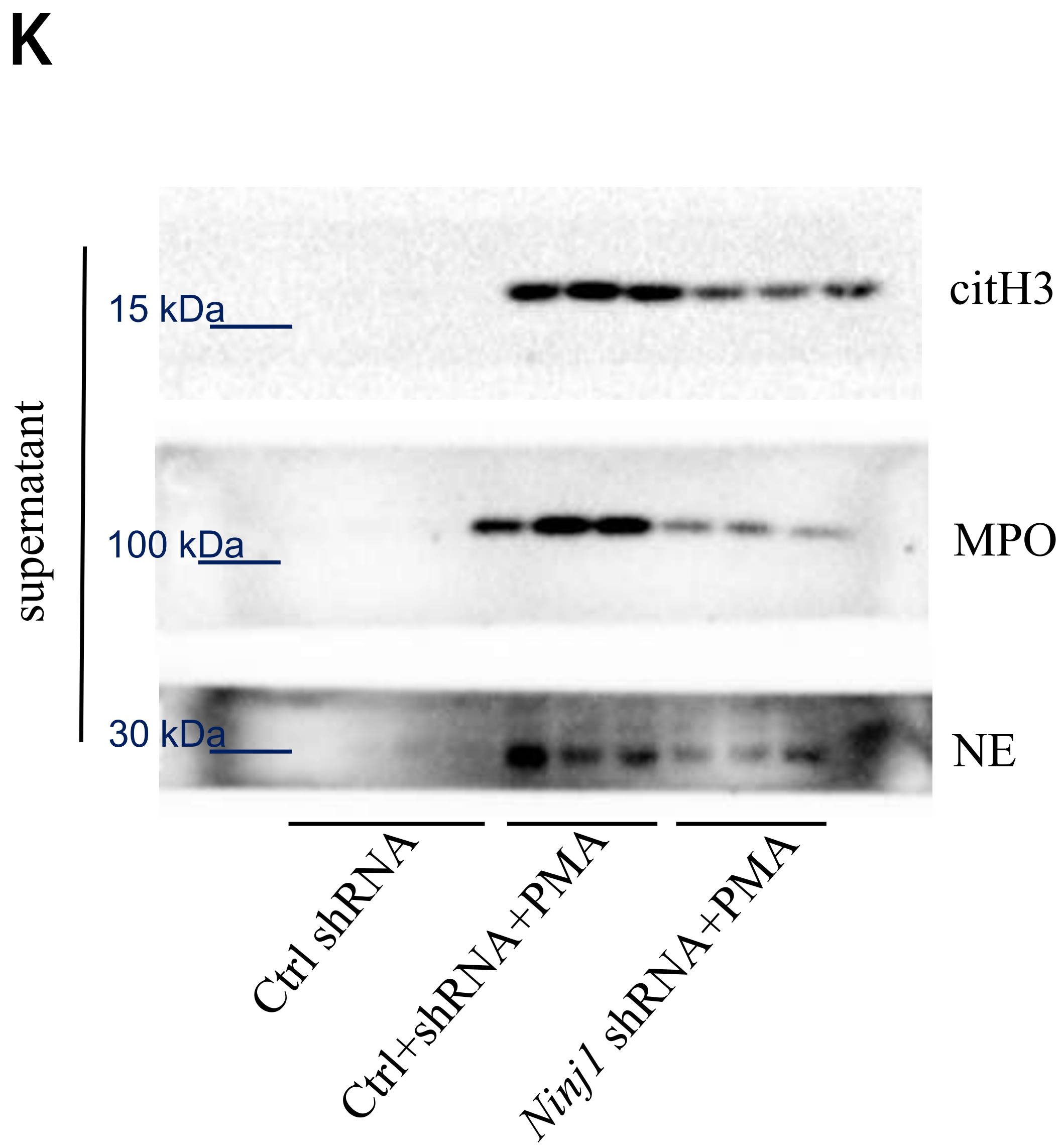
K
supernatant
15 kDa
100 kDa
30 kDa
citH3
MPO
NE
Ctrl shRNA
Ctrl+shRNA+PMA
Ninj1 shRNA+PMA

Figure 7

E

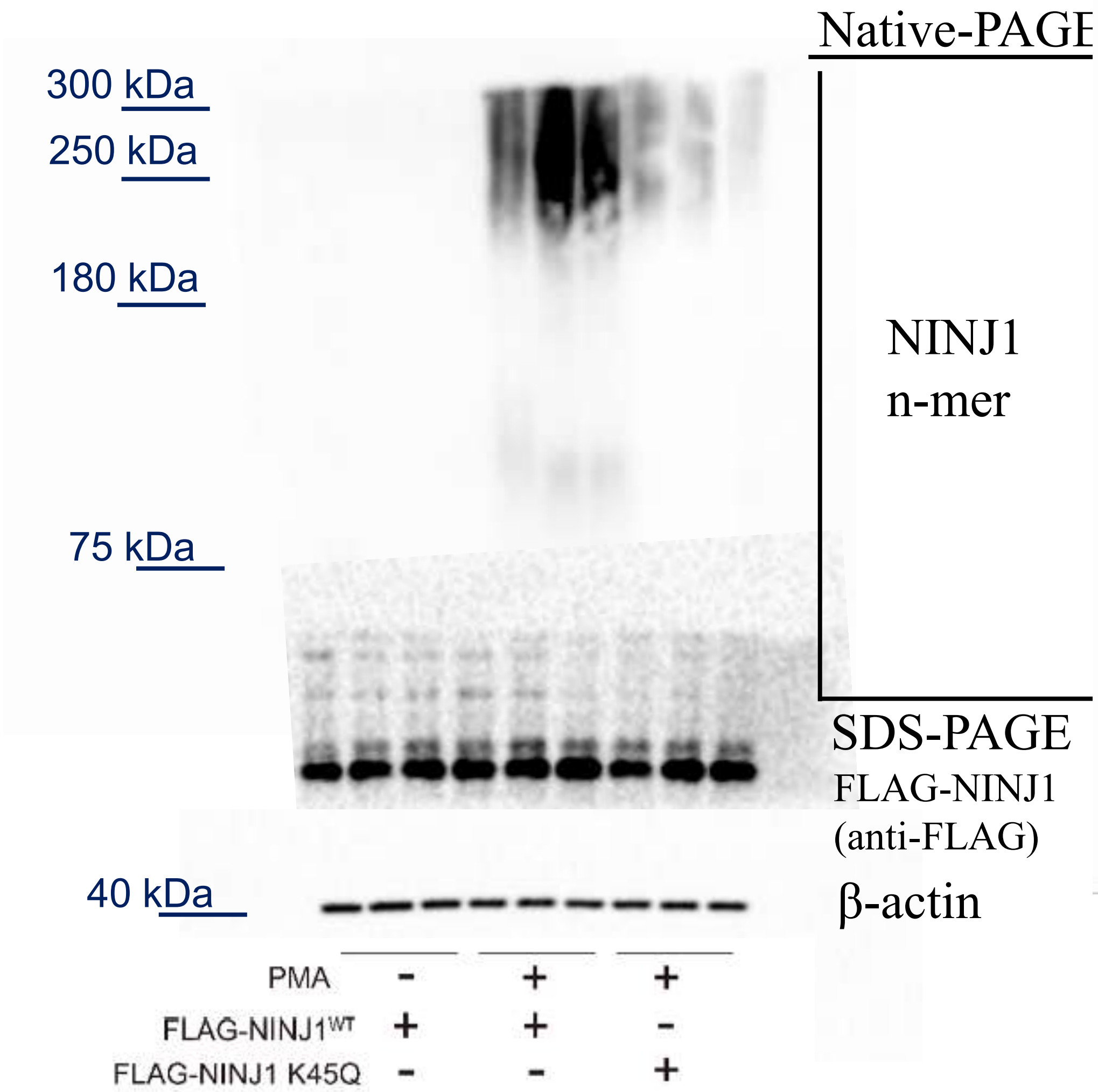
300 kDa
250 kDa
180 kDa
75 kDa
40 kDa
Native-PAGE
NINJ1
n-mer
SDS-PAGE
FLAG-NINJ1
(anti-FLAG)
β-actin
PMA - + +
FLAG-NINJ1WT + + -
FLAG-NINJ1 K45Q - - +

F

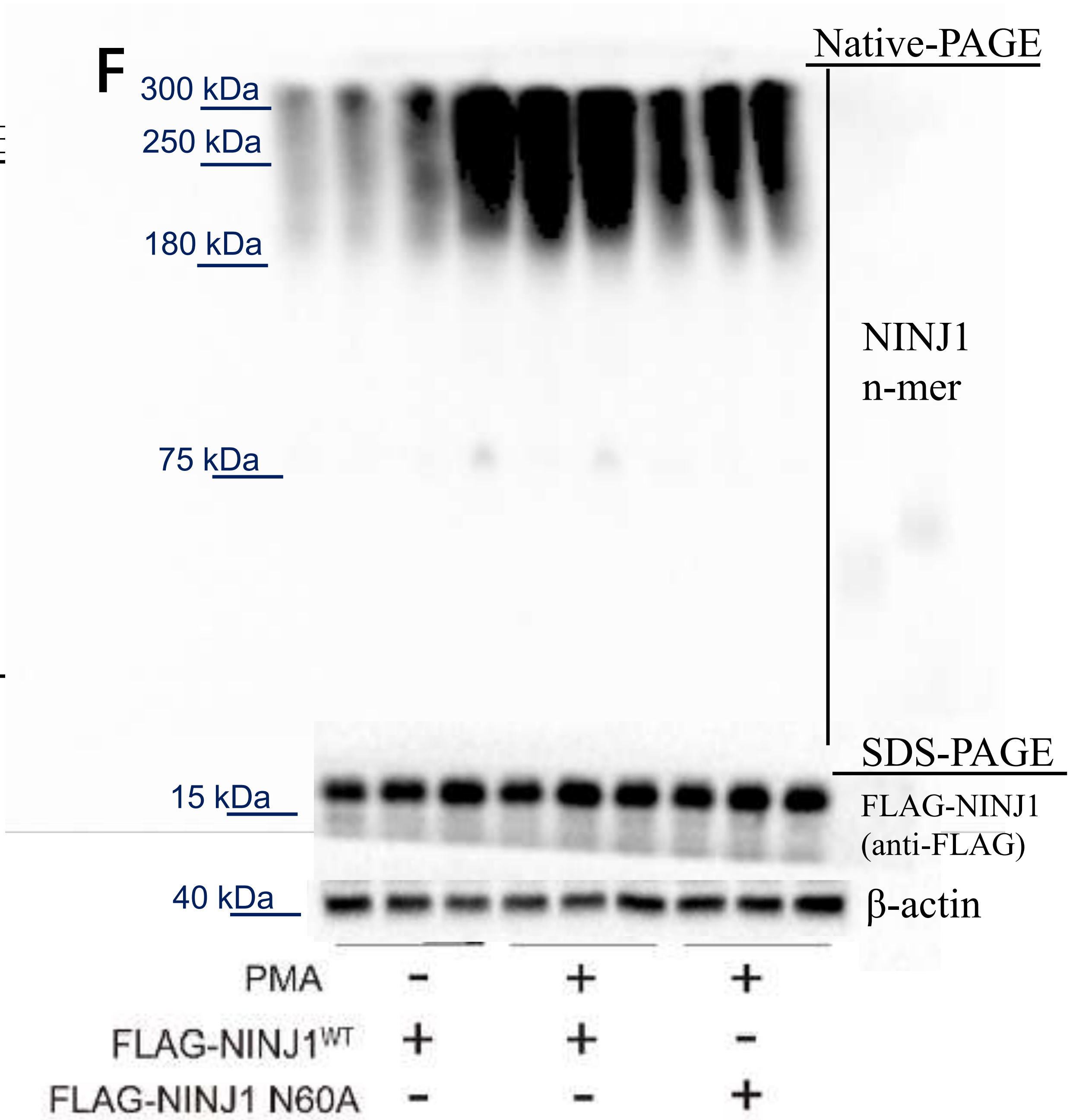
300 kDa
250 kDa
180 kDa
75 kDa
15 kDa
40 kDa
Native-PAGE
NINJ1
n-mer
SDS-PAGE
FLAG-NINJ1
(anti-FLAG)
β-actin
PMA - + +
FLAG-NINJ1WT + + -
FLAG-NINJ1 N60A - - +

Figure 7

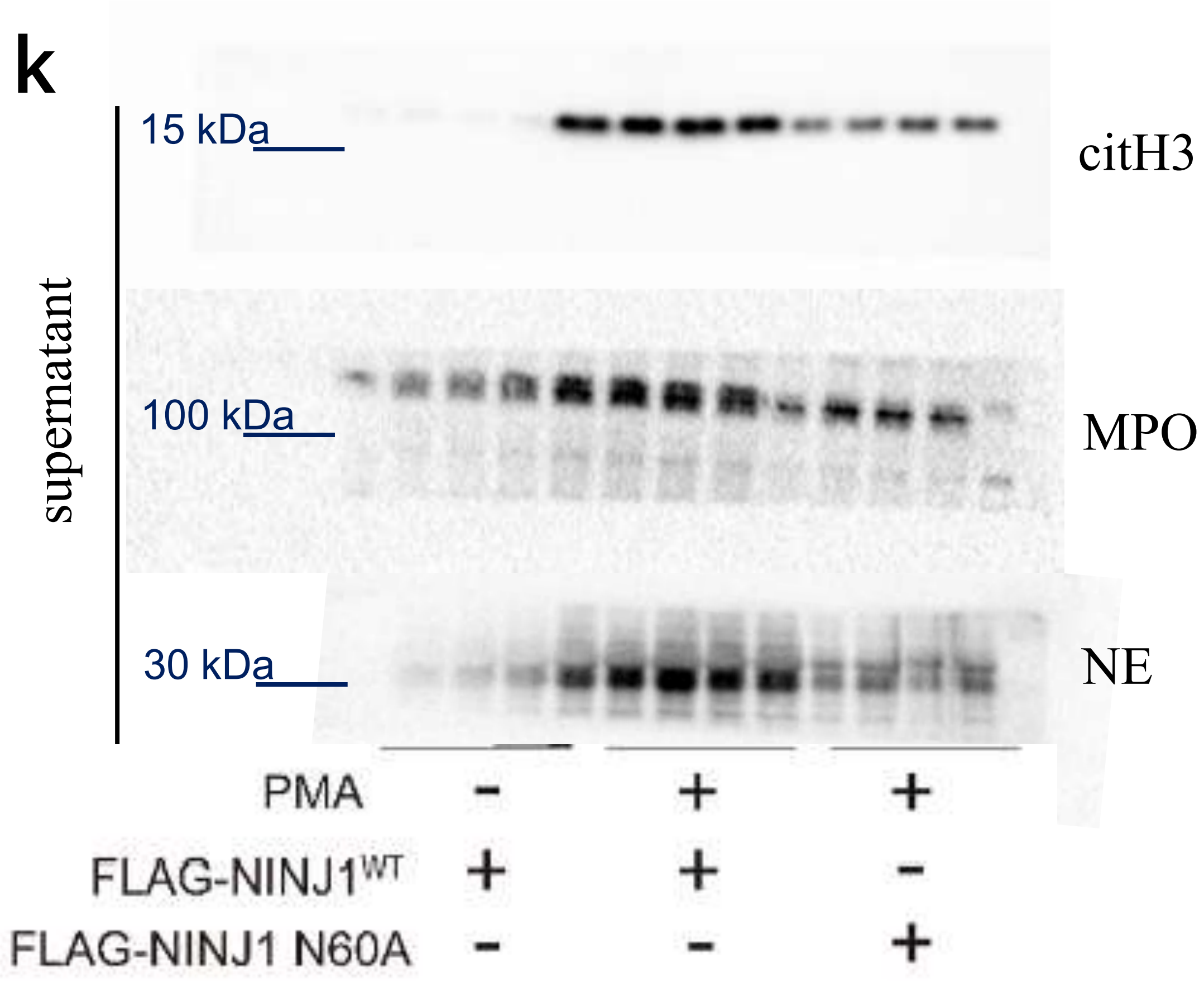
k
15 kDa
citH3
100 kDa
MPO
30 kDa
NE
supernatant
PMA - + +
FLAG-NINJ1WT + + -
FLAG-NINJ1 N60A - - +

Supplementary figure 4

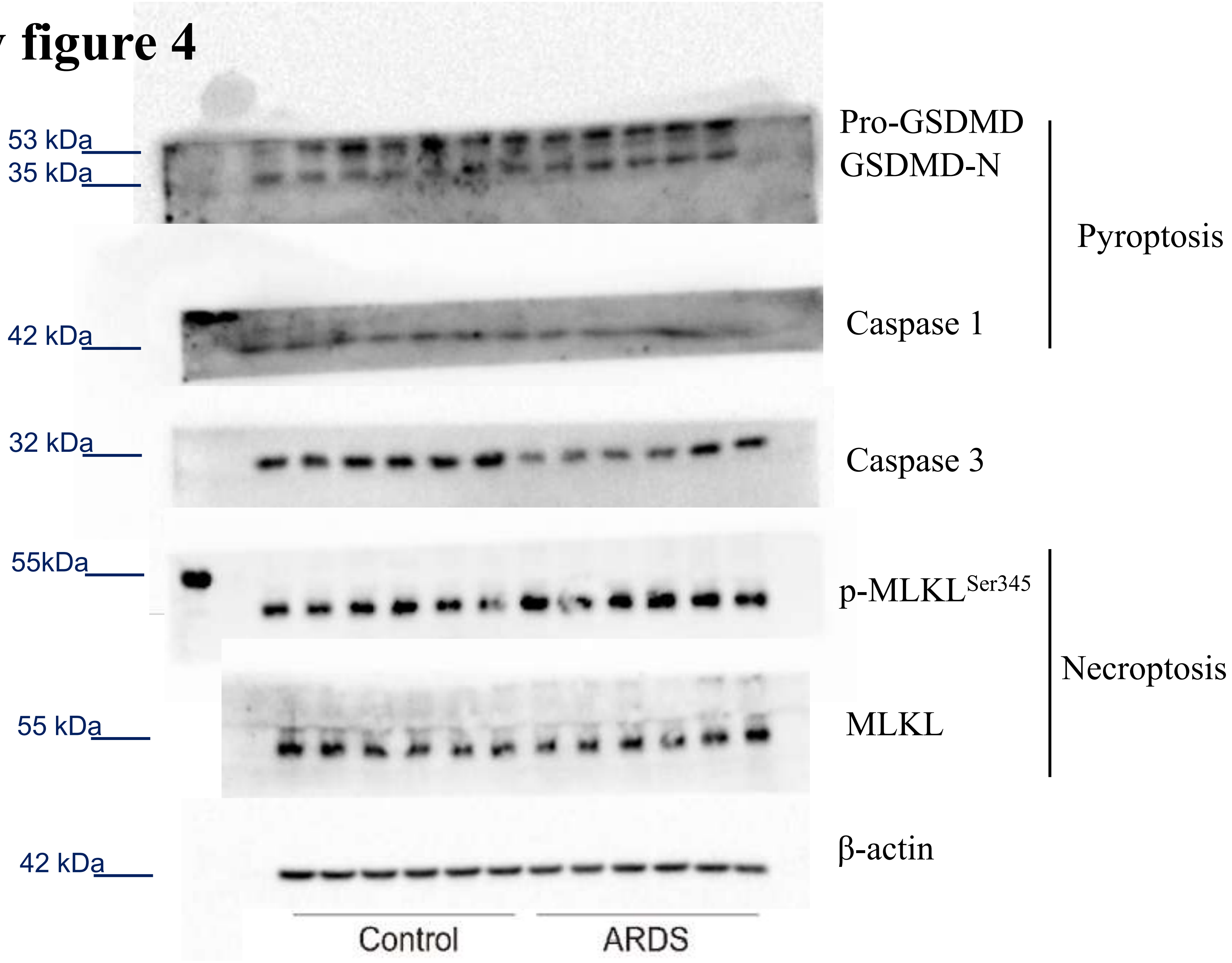

Supplementary figure 5

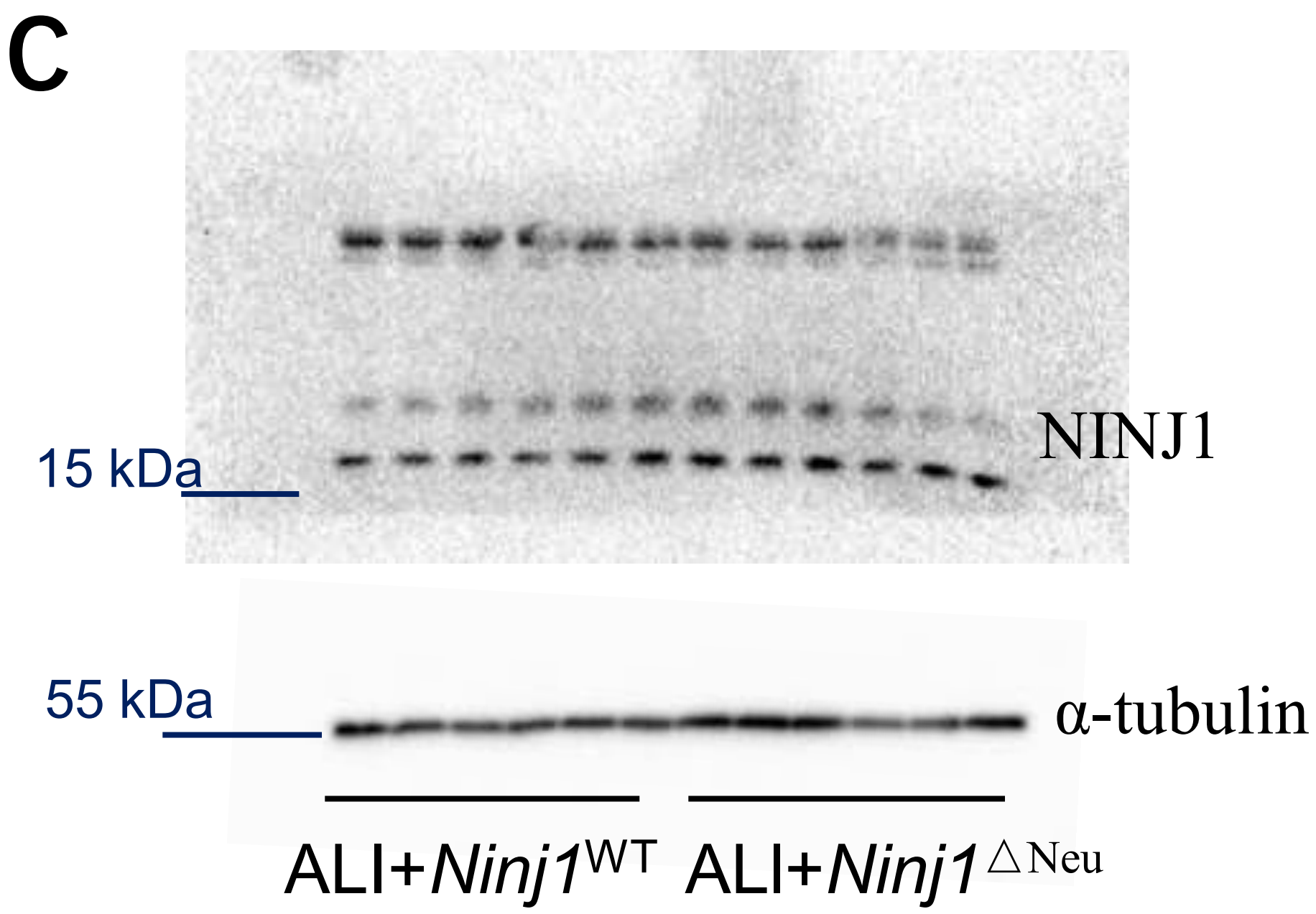

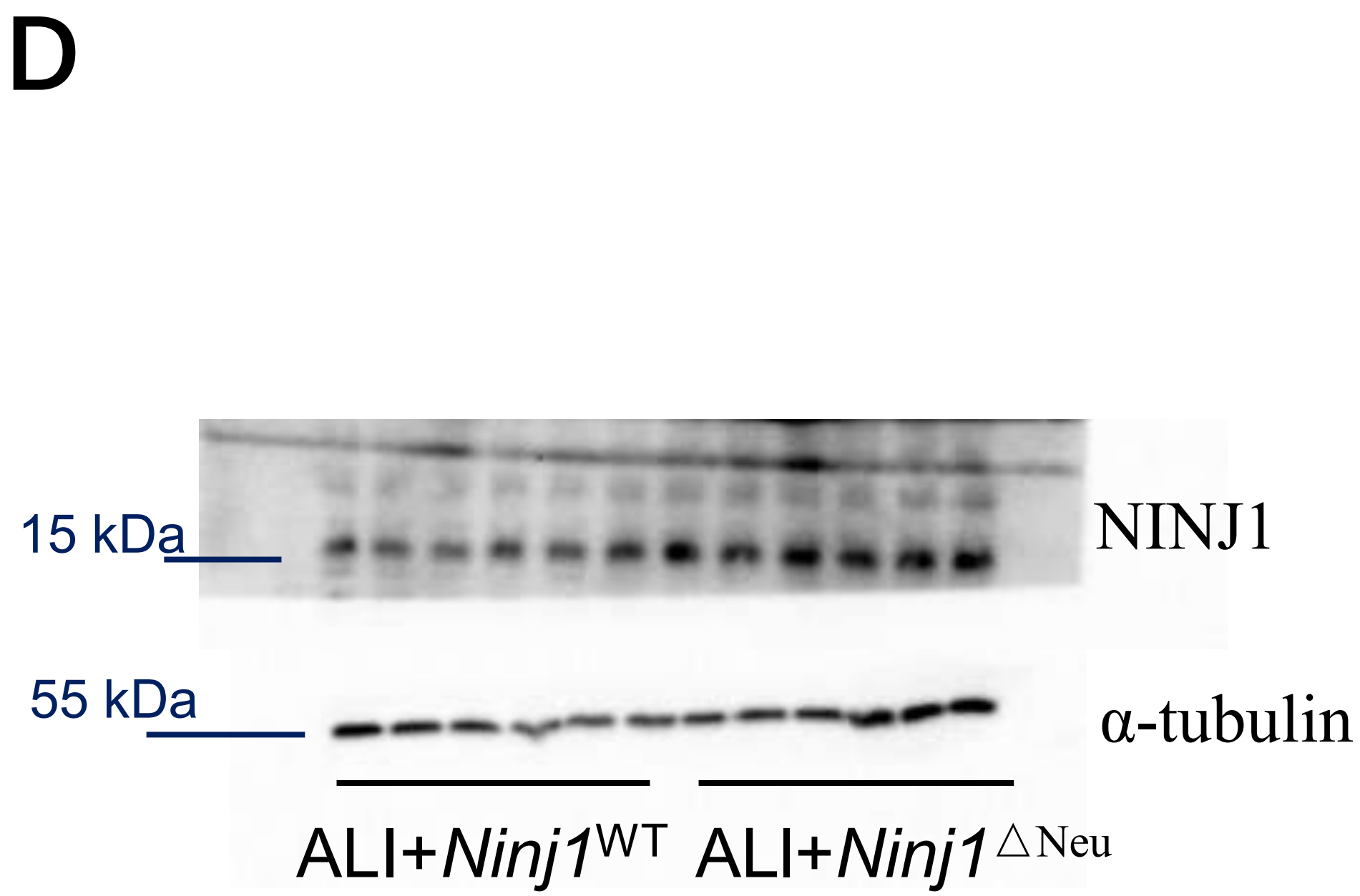

Supplementary figure 10

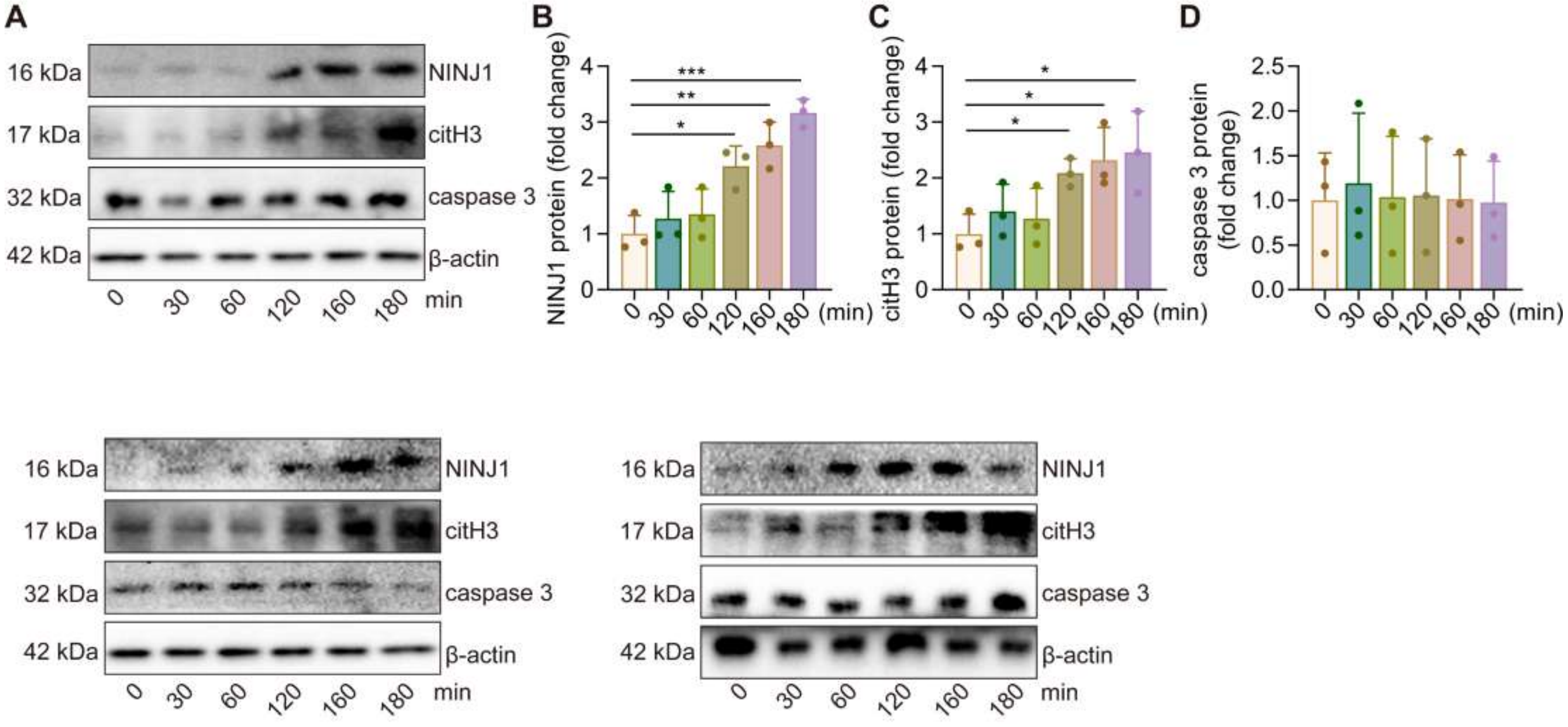

Supplementary figure 11

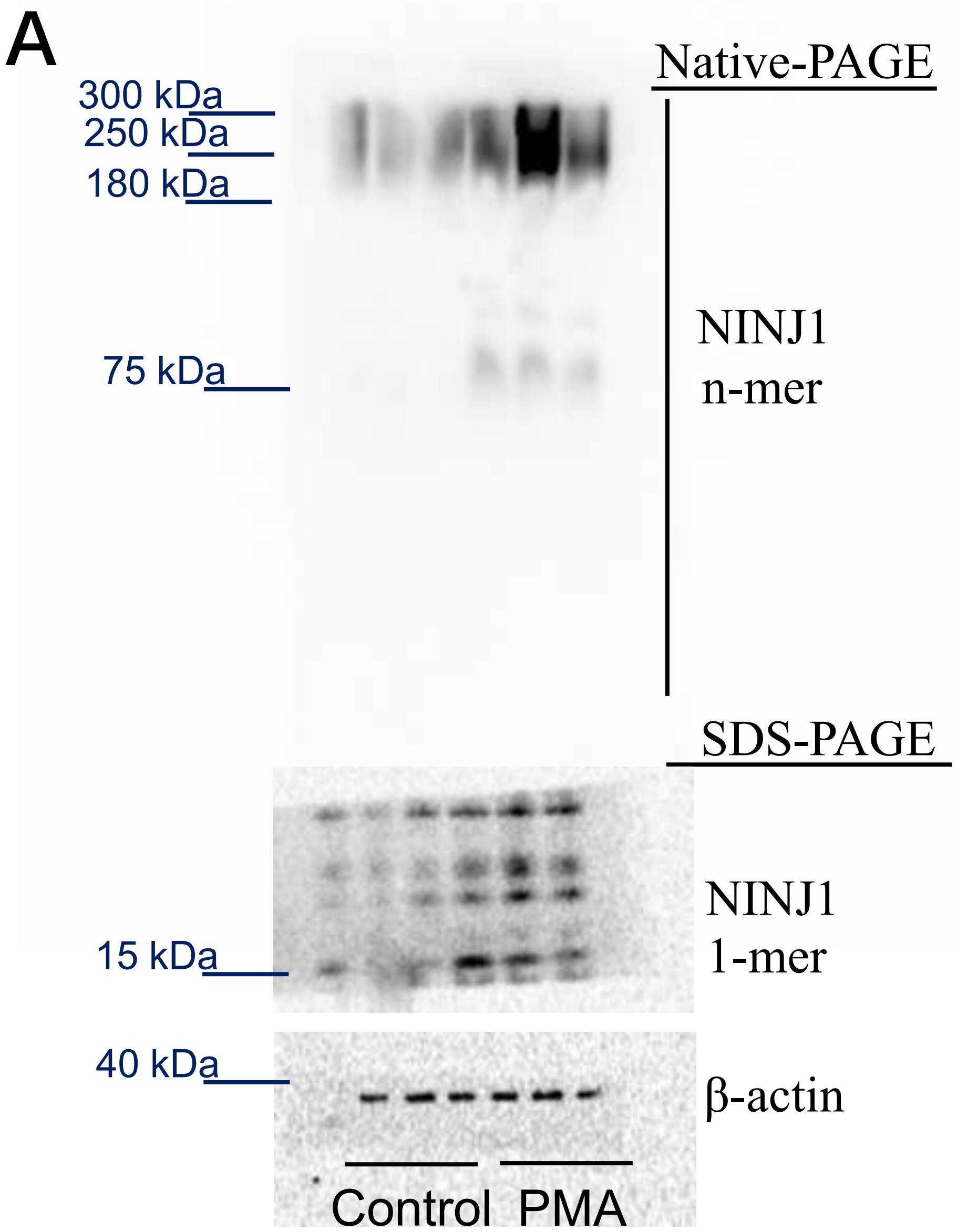

Supplementary figure 12

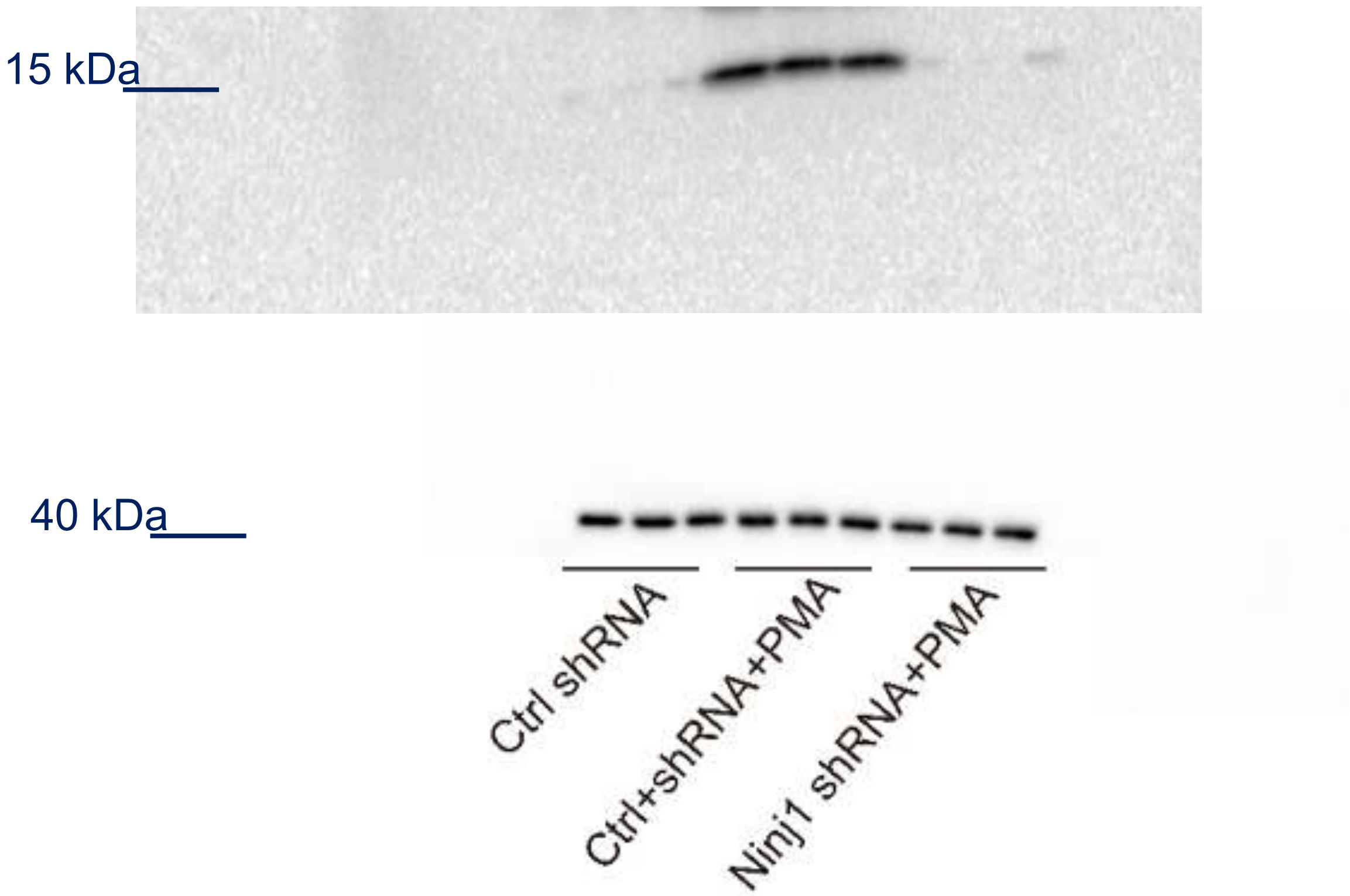

Supplementary figure 13

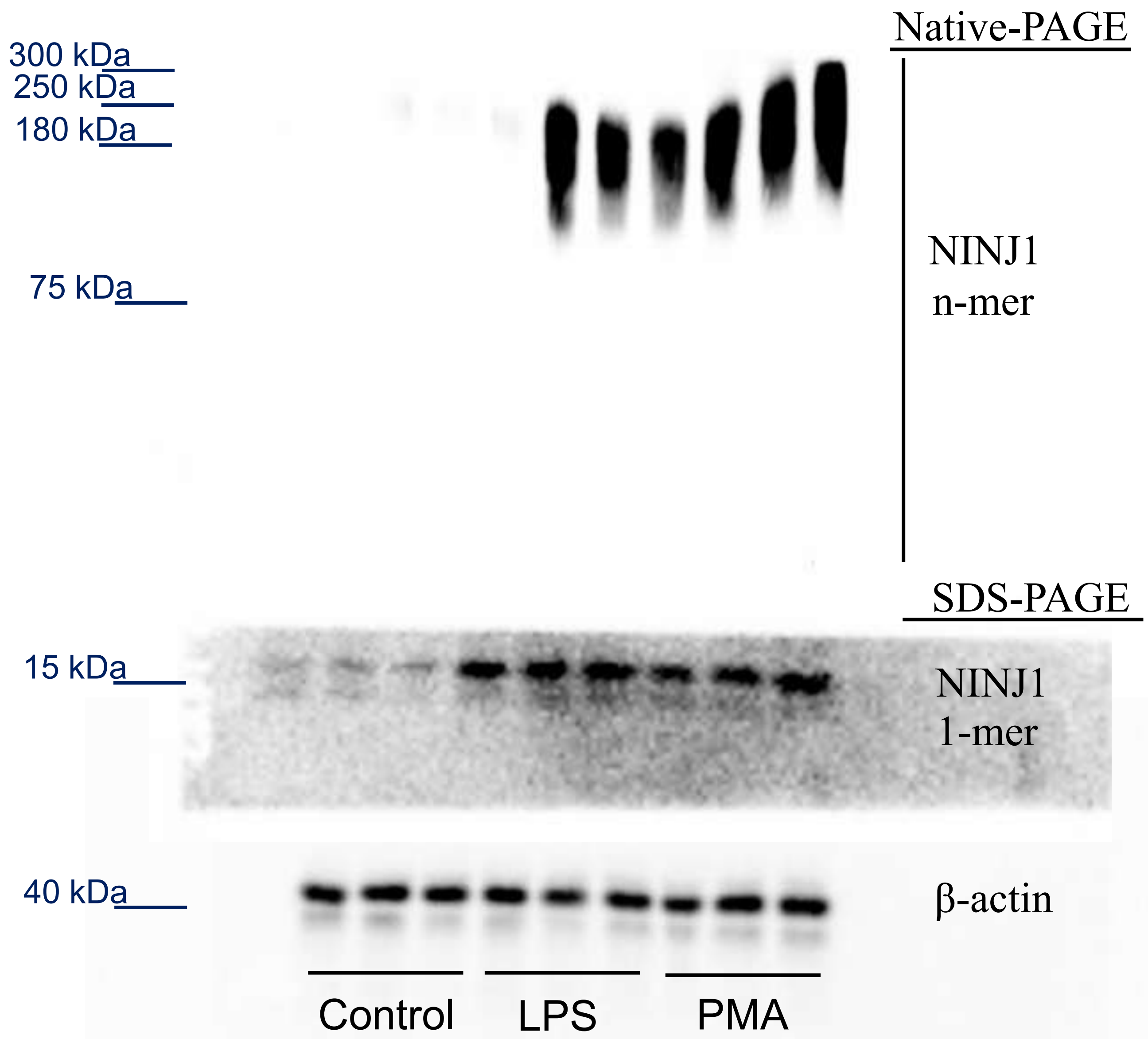

Supplementary figure 14

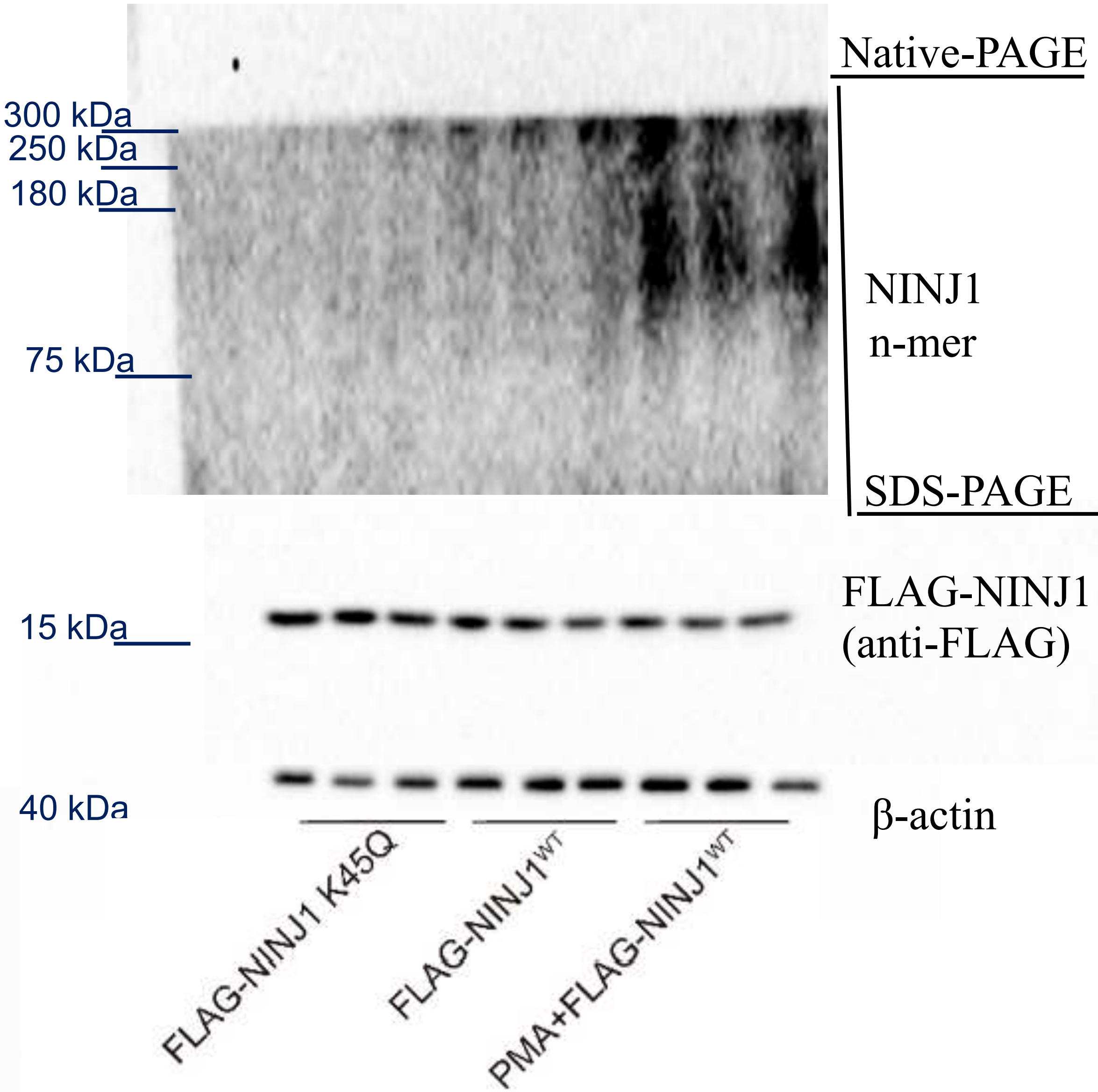

Supplementary figure 15

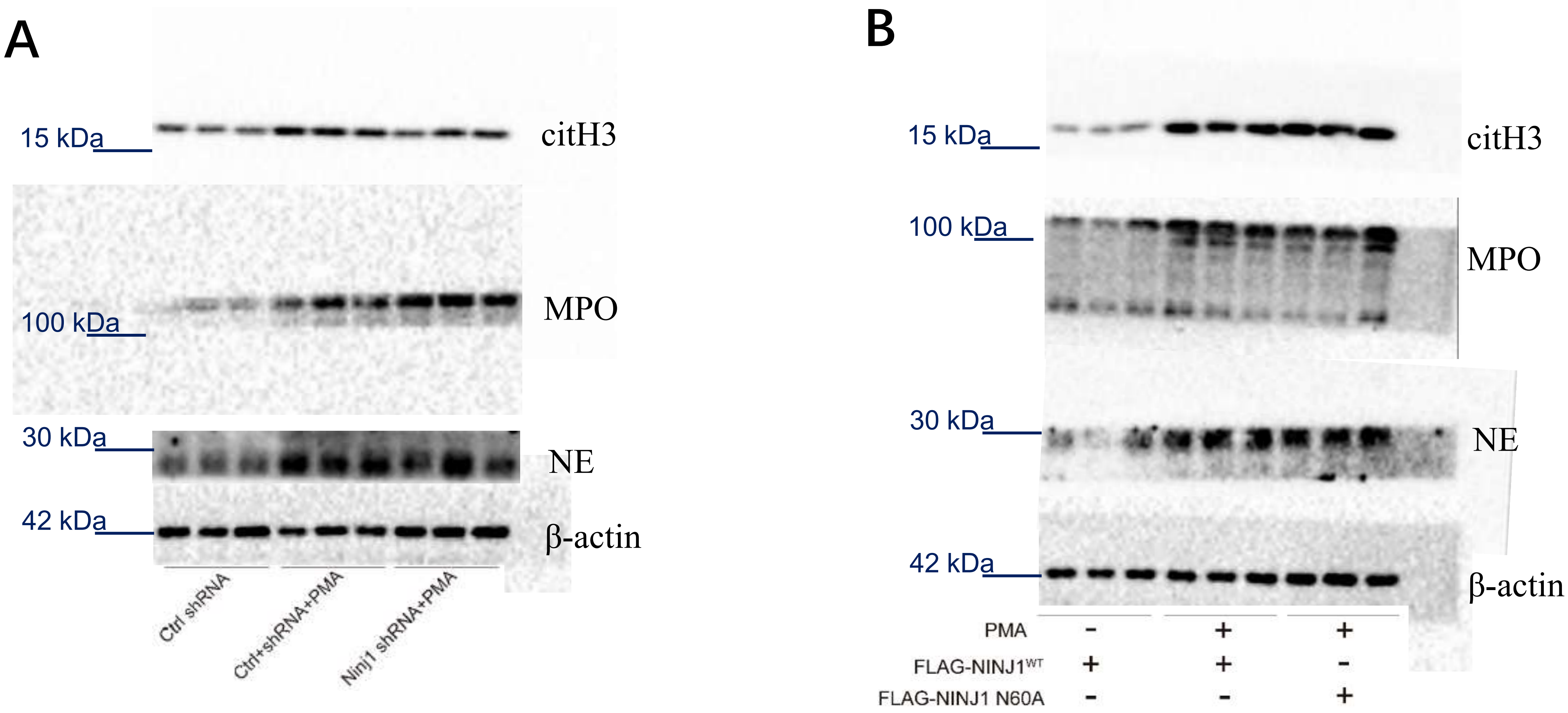

Supplementary figure 16

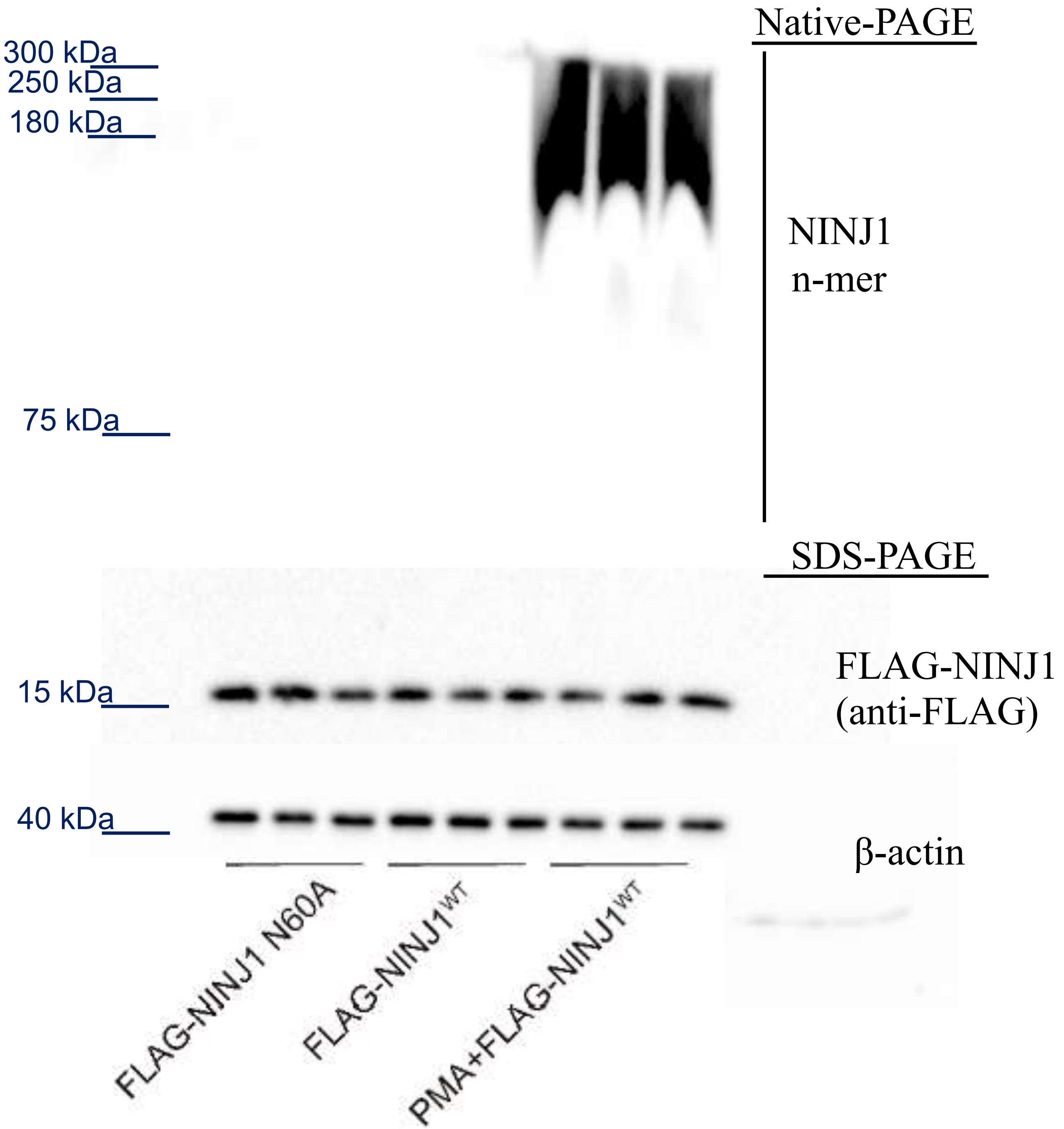

Supplement: Supplementary file 1 [file 41419_2026_8995_MOESM1_ESM.pdf]
