# Supplementary material for: NINJ1 plays a vital role in the release of neutrophil extracellular traps during acute lung injury
Source: Cell Death Dis. 2026 Jun 25;17(1):787. doi: 10.1038/s41419-026-08995-5 (PMC13558649; doi:10.1038/s41419-026-08995-5)
Supplement: Supplementary file 2 [file 41419_2026_8995_MOESM4_ESM.pdf]

Corresponding Author Name: Yong Zhou, Jia
Manuscript Number: CDDIS-25-827

SPRINGER NATURE

Reporting Summary

Springer Nature wishes to improve the reproducibility of the work that we publish. This checklist is used to ensure good reporting standards and to improve the reproducibility. Please respond completely to all questions relevant to your manuscript. For more information, please read the journal's Guide to Authors.

☑ **Check here to confirm that the following information is available in the Material & Methods section:**

- **The exact sample size (*n*)** for each experimental group/condition, given as a number, not a range
- **A description of the sample collection** allowing the reader to understand whether the samples represent technical or biological replicates (including how many animals, litters, culture, etc.)
- **A statement of how many times the experiment shown was replicated in the laboratory**
- **Definitions of statistical methods and measures**: For small sample sizes (n<5) descriptive statistics are not appropriate, instead plot individual data points
  - Very common tests, such as *t*-test, simple χ^2 tests, Wilcoxon and Mann-Whitney tests, can be unambiguously identified by name only, but more complex techniques should be described in the methods section
  - Are tests one-sided or two-sided?
  - Are there adjustments for multiple comparisons?
  - **Statistical test results**, e.g., ***P* values**
  - Definition of **'center values'** as **median or mean**;
  - Definition of **error bars as s.d. or s.e.m. or c.i.**

Please ensure that the answers to the following questions are reported in the manuscript itself. We encourage you to include a specific subsection in the methods section for statistics, reagents and animal models. Below, provide the page number or section and paragraph number.

Statistics and general methods

	**Reported in section/paragraph or page #**
1. How was the sample size chosen to ensure adequate power to detect a pre-specified effect size? (Give section/paragraph or page #)	No prospective statistical method was used to predetermine sample size. The mouse sample size (n = 6 per group) was chosen based on preliminary experiments
For animal studies, include a statement about sample size estimate even if no statistical methods were used.	Methods, Page 20, "Statistical analysis"
2. Describe inclusion/exclusion criteria if samples or animals were excluded from the analysis. Were the criteria pre-established? (Give section/paragraph or page #)	Inclusion/exclusion criteria for human ARDS patients and healthy controls are described in the Methods section (page 15, "Recruitment of healthy controls and clinical ARDS patients"). Exclusion criteria included pregnancy, age
	Mice with unsuccessful intratracheal instillation (e.g., immediate reflux of the instilled solution, or death during or immediately after the procedure) were excluded from the analysis. These criteria were pre-established before the
3. If a method of randomization was used to determine how samples/animals were allocated to experimental groups and processed, describe it. (Give section/paragraph or page #)	
For animal studies, include a statement about randomization even if no randomization was used.	Mice were randomly allocated to experimental groups using a computer-generated randomization schedule. This

4. If the investigator was blinded to the group allocation during the experiment and/or when assessing the outcome, state the extent of blinding. (Give section/paragraph or page #)	Investigators were blinded to group allocation during outcome assessment, particularly for histopathological scoring, micro-CT analysis, and imaging quantification. This is stated in the Methods section (page 11, "Lung histology
For animal studies, include a statement about blinding even if no blinding was done.	the Methods section, page 11, "Lung histology and inflammatory injury score".
5. For every figure, are statistical tests justified as appropriate?	Yes. Statistical justification is provided in the Methods section (page 20, 'Statistical analysis'). Additionally, each
Do the data meet the assumptions of the tests (e.g., normal distribution)?	Yes. in the Methods section, page 20, 'Statistical analysis'
Is there an estimate of variation within each group of data?	Yes. in the Methods section, page 20, 'Statistical analysis'
Is the variance similar between the groups that are being statistically compared? (Give section/paragraph or page #)	Yes. in the Methods section, page 20, 'Statistical analysis'

Reagents

Reported in section/paragraph or page #

6. Report the source of antibodies (vendor and catalog number)	All antibody sources and catalog numbers are provided in Table 2 (page 43).
7. Identify the source of cell lines and report if they were recently authenticated (e.g., by STR profiling) and tested for mycoplasma contamination	The dHL-60 cell line was obtained from ATCC (RRID: CVCL_HL60). Cells were authenticated by STR profiling and tested negative for mycoplasma contamination. This information is provided in the Methods section (page 17,

Animal Models

Reported in section/paragraph or page #

8. Report species, strain, sex and age of animals	Adult male C57BL/6J mice, 8–10 weeks of age, 20–25 g.
9. For experiments involving live vertebrates, include a statement of compliance with ethical regulations and identify the committee(s) approving the experiments.	All animal experiments were approved by the IRB of the Xiangya School of Basic Medical Science at Central South University (Approval No. 2023-KT107). This is stated in the Methods section (page 7, "Ethics statement"

10. We recommend consulting the ARRIVE guidelines (*PLoS Biol.* **8**(6), e1000412,2010) to ensure that other relevant aspects of animal studies are adequately reported.

Human subjects

	Reported in section/paragraph or page #
11. Identify the committee(s) approving the study protocol.	The Clinical Medical Ethics Committee of Xiangya Hospital, Central South University approved the human study
12. Include a statement confirming that informed consent was obtained from all subjects.	Informed consent was obtained from all patients and healthy controls. This is stated in the Methods section (page 7, "Ethics
13. For publication of patient photos, include a statement confirming that consent to publish was obtained.	Not applicable. No patient photos are included in this manuscript.
14. Report the clinical trial registration number (at ClinicalTrials.gov or equivalent).	Not applicable. This study is not a clinical trial.

15. For phase II and III randomized controlled trials, please refer to the CONSORT statement and submit the CONSORT checklist with your submission.

16. For tumor marker prognostic studies, we recommend that you follow the REMARK reporting guidelines.

Data deposition

	Reported in section/paragraph or page #
17. Provide accession codes for deposited data. Data deposition in a public repository is mandatory for:<br>a. Protein, DNA and RNA sequences<br>b. Macromolecular structures<br>c. Crystallographic data for small molecules<br>d. Microarray data	Publicly available datasets used include GSE224938, GSE36809070, GSE203340, and GSE157789 This is stated in the Methods section (page 12–13) and figures.

Deposition is strongly recommended for many other datasets for which structured public repositories exist; more details on our data policy are available in the Guide to Authors. We encourage the provision of other source data in supplementary information or in unstructured repositories such as Figshare and Dryad. We encourage publication of Data Descriptors (see Scientific Data) to maximize data reuse.

18. If computer code was used to generate results that are central to the paper's conclusions, include a statement in the Methods section under "**Code availability**" to indicate whether and how the code can be accessed. Include version information as necessary and any restrictions on availability.	No custom computer code was generated for this study. All bioinformatics analyses were performed using publicly available software as described in the Methods section (e.g., Seurat v5, Monocle2, Hotspot v0.9.1).
