# Supplementary material for: NINJ1 plays a vital role in the release of neutrophil extracellular traps during acute lung injury
Source: Cell Death Dis. 2026 Jun 25;17(1):787. doi: 10.1038/s41419-026-08995-5 (PMC13558649; doi:10.1038/s41419-026-08995-5)
Supplement: Supplementary file 3 [file 41419_2026_8995_MOESM3_ESM.docx]

**NINJ1 plays a vital role in the release of neutrophil extracellular traps in acute lung injury**

Wen-Jing Zhong^1,2,3^, Yu-Biao Liu^2,3,4^, Xin-Yu Yang^2,3,4^, Meng-Rui Chen^2,3,4^, Nan-Shi-Yu Yang^2,3,4^, Chen-Yu Zhang^2,3,4^, Jian-Bing Xiong^5,6^, Wei-Feng Tang^7^, Cha-Xiang Guan^2,3,8^, Yan-Feng Zhang^1,6^, Jia-Xi Duan*^1,6^, Yong Zhou*^2,3,4^

^1^ Department of Geriatric Respiratory and Critical Care Medicine, Xiangya Hospital, Central South University, Changsha, Hunan 410008, China;

^2^ Department of Physiology, Xiangya School of Basic Medical Sciences, Central South University, Changsha, Hunan 410078, China;

^3^ National Experimental Teaching Demonstration Center for Medical Function, Central South University, Changsha, Hunan 410078, China;

^4^ Key Laboratory of General University of Hunan Province, Basic and Clinical Research in Major Respiratory Disease, Changsha, Hunan 410078, China;

^5^ Department of Emergency, Xiangya Hospital, Central South University, Changsha, Hunan 410078, China;

^6^ National Clinical Research Center of Geriatric Disorders, Xiangya Hospital, Central South University, Changsha, Hunan 410008, China;

^7^ Department of Pulmonary and Critical Care Medicine, The Second Xiangya Hospital, Central South University, Changsha, Hunan 410011, China;

^8^ School of Basic Medicine, Ningxia Medical University, Yinchuan, Ningxia 750004, China.

Keywords: Ninjurin-1, acute lung injury, neutrophils, neutrophil extracellular traps

***To whom correspondence should be addressed:**

Prof. Yong Zhou

Department of Physiology

Xiangya School of Basic Medical Sciences, Central South University

Changsha, Hunan 410078, China

Tel.: +86-731-88660446

E-mail: zhouyong421@csu.edu.cn

or

Dr. Jia-Xi Duan

Department of Geriatric Respiratory and Critical Care Medicine, Xiangya Hospital, Central South University

Changsha, Hunan 410008, China

E-mail: duanjiaxi91@csu.edu.cn

**Supplementary Materials**

**Supplementary Table**

Supplementary Table 1. Antibody sources and dilutions.

Supplementary Table 2. Highly Conserved Ninj1 Sequences in Vertebrates.

**Supplementary Figures**

Supplementary Figure 1. NINJ1 expression is significantly increased in ALI/ARDS.

Supplementary Figure 2. Single-cell RNA-seq analysis of peripheral blood neutrophils in ARDS patients.

Supplementary Figure 3. Increased NINJ1 expression in ARDS patients by flow cytometry.

Supplementary Figure 4. No alterations in apoptosis, necroptosis, or pyroptosis of neutrophils from ARDS patients.

Supplementary Figure 5. Generation of neutrophil-specific conditional *Ninj1*-null mice.

Supplementary Figure 6. Normal lung histology and micro-CT in unstimulated neutrophil-specific *Ninj1-*depleted mice.

Supplementary Figure 7. Neutrophil-specific *Ninj1* depletion reduces neutrophil infiltration.

Supplementary Figure 8. Validation of successful HL‑60 cell differentiation by Giemsa staining.

Supplementary Figure 9. Real-time reveals multiple pore‑forming proteins in LPS‑primed mouse neutrophils undergoing NETosis.

Supplementary Figure 10. Time‑dependent formation of NETosis and upregulation of NINJ1 in the absence of apoptosis in PMA‑stimulated mouse neutrophils.

Supplementary Figure 11. NINJ1 oligomerization is elevated in PMA-induced neutrophils.

Supplementary Figure 12. Validation of Ninj1 knockdown and inhibition of PMA‑induced NETs upon NINJ1 silencing.

Supplementary Figure 13. Ninj1 knockdown prevents PMR in LPS-treated neutrophils.

Supplementary Figure 14. No effect of untreated K45Q on neutrophil NINJ1 oligomerization and NETs release.

Supplementary Figure 15. No alteration in NETosis formation in Ninj1‑silenced and NINJ1‑N60A mutant neutrophils.

Supplementary Figure 16. No effect of untreated N60A on neutrophil NINJ1 oligomerization and NETs release.

Supplementary Figure 17. Lack of upregulated NINJ1 phosphorylation upon PMA stimulation.

**Supplementary Tables**

Supplementary Table 1. Antibody sources and dilutions.

| Antibodies | Source | Catalog | Dilution ratio |
| --- | --- | --- | --- |
| Primary antibodies for Western blot | | | |
| Rabbit anti-MLKL polyclonal antibody | Abcam | Ab172868 | 1:2000 |
| Rabbit anti-phospho-MLKL^S345^ antibody | Abcam | Ab196436 | 1:2000 |
| Rabbit-anti-caspase3 antibody | CST | 9662 | 1:2000 |
| Anti-GSDMD monoclonal antibody | Santa Cruz | sc-393581 | 1:1000 |
| Anti-caspase1/p10/p20 monoclonal antibody | Abcam | Ab179515 | 1:1000 |

Supplementary Table 2. Highly Conserved Ninj1 Sequences in Vertebrates.

| **Scientific name** | **Sequence** |
| --- | --- |
| NINJ1_HUMAN  NINJI_MOUSE  NINJ1_RAT  NINJI_PIG  NINJ1_GOAT  CONS | MDSGTEEYELNGGLPPGTPGSPDASPARWGWRHGPINVNHYASKKSAAESMLDIALLMANASQLKAWE  MESGTEEYELNGDLRPGSPGSPDALPPRWGLRNRPINVNHYANKKSAAESMLDIALLMANASQLKAVVE  MDPGTEEYELNGDLRPGSPGSPDASPPRWGLRNRPINVNHYANKKSAAESMLDIALLMANASQLKAVVE  MDSRLEDYELNGDLRPVSPSSPDASPPRWG - RNRPINMNHYANKKSAAESMLDIALLMANASQLKAVIE  MDSRPE EYELNGDLRPS SPGSPDASPSRWG - RNQPINMNHYANKKSAAESMLDIALLMANASQLKAVIE  * : . * : * * * * * . * * : * . * * * * * . * * * * : *** : * * * * . * * * * * * ** * * * *** * * * * * ** * ** : * |
| NINJ1_HUMAN  NINJI_MOUSE  NINJ1_RAT  NINJI_PIG  NINJ1_GOAT  CONS | QGPSFAFYVPLVVLISISLVLQIGVGVLLIFLVKYDLNNPAKHAKLDFLNNLATGLVFIIVVVNIFITA  QGNDFAFFVPLVVLISISLVLQIGVGVLLIFLVKYDLNNPAKHAKLDFLNNLATGLVFIIVVVNIFITA  QGNEFAFFVPLVVLISISLVLQIGVGVLLIFLVKYDLNNPAKHAKLDFLNNLATGLVFIIVVVNIFITA  QG PSFAFFIPLVVLISISLVLOV GVGVLLIFLVRYDLNNPAKHAKLDFLNNLATGLVFIIVVVNIFITA  QG TGFAFFIPLVVLISISLVLQ I GVGVLLIFLVRYDLNNPAKHAKLDFLNNLATGLVFIIVVVNIFITA  * * ***: : ** * ** ****** * * : * * * ** * *** * . * * * * * ** * * * * * ** * * * * * * * * ***** * * * ***** |
| NINJ1_HUMAN  NINJI_MOUSE  NINJ1_RAT  NINJI_PIG  NINJ1_GOAT  CONS | FGVQKPLMDMAPQQ  FGVQKPVMDVAPRQ  FGVQKPVMDVAPRQ  FGVQKPMMDVAPRQ  FGVQKP T MDTAPRQ  * * * * * * ** ** . * |

Note: Amino acid sequences of Ninj1 in various species of vertebrates were collected from UniProt knowledgebase and were aligned by T-Coffee. Then, highly conserved sequences are listed. N-glycosylation motifs are underlined, and an essential Asn in each species is marked in red. Identical amino acids are marked with an asterisk.

**Supplementary Figures**

**Figure S1.** **NINJ1 expression is significantly increased in ALI/ARDS.**

A) Human *NINJ1* mRNA expression in the lungs from ARDS patients was determined by microarray (GSE32707) (*n*=14). Lung tissue samples were collected from enrolled ARDS patients at the time of hospital admission (Day 0) and on Day 7 thereafter. The Control group consisted of patients undergoing mechanical ventilation without sepsis, SIRS, or ARDS. B) *Ninj1* mRNA expression in the lungs from LPS-induced ALI mice (GSE203340), *n*=3. Data were shown as mean ± SD. ****P* < 0.001 (unpaired two‑tailed Student's t‑test).

**Figure S2. Single-cell RNA-seq analysis of peripheral blood neutrophils in ARDS patients.**

A) Distribution of 7 subtypes of cells by UMAP, coloured by cell types (GSE157789). t-SNE visualizations depict Ninj1 expression levels, defined by binarized activity thresholds derived from AUC scores. B) The expression of NINJ1 over all peripheral blood cells, with each point from an individual sample.

**Figure S3. Increased NINJ1 expression in ARDS patients by flow cytometry.**

Flow cytometric analysis of live (PI⁻) neutrophils expressing NINJ1 in ARDS patients versus Controls.

**Figure S4**. **No alterations in pyroptosis, apoptosis, or necroptosis of neutrophils from ARDS patients.**

SDS‑PAGE analysis of GSDMD, caspase 1, caspase 3, and MLKL in peripheral neutrophils from Healthy Controls and ARDS patients. β‑actin was used as the loading Control, *n*=6.

**Figure S5. Generation of neutrophil-specific conditional *Ninj1*-null mice.**

A) Targeted disruption of the *Ninj1* gene was verified by PCR of genomic DNA isolated from candidate mice. B) Targeted disruption of the *Ly6G*^Cre^ gene was verified by PCR of genomic DNA isolated from candidate mice. *Ninj1*^WT^ and *Ninj1*^ΔNeu^ mice were intratracheally administered with LPS (5 mg/kg). C) Real-time PCR and Western blot were employed to detect NINJ1 expression in Ly6G^-^ cells isolated from bone marrow from *Ninj1*^WT^ and *Ninj1*^ΔNeu^ mice. D) Detection of NINJ1 expression by Real-time PCR and Western blot in BMDMs from *Ninj1*^WT^ and *Ninj1*^ΔNeu^ mice. E) The physical appearance of *Ninj1*^WT^ and *Ninj1*^ΔNeu^ mice administered with LPS, *n* =3-6. Data were shown as mean ± SD. Unpaired two‑tailed Student's *t*‑test.

**Figure S6. Lung histology and micro-CT in unstimulated neutrophil-specific *Ninj1*-null mice.**

A) High-resolution micro-CT was utilized to assess pulmonary structural changes in unstimulated neutrophil-specific *Ninj1*-depleted mice. Multiplanar reconstructions, including dorsal and axial views, were obtained at the level of the T6 thoracic vertebra. Representative 3D reconstructions were generated from *in vivo* acquired images, with voxel intensities thresholded within a Hounsfield unit (HU) range of -1000 to -400. B) Representative images of the mouse lung tissue (Left panel) and the lung histopathology stained with H&E (Right panel), scale bar = 50 μm.

**Figure S7. Neutrophil-specific *Ninj1* depletion reduces neutrophil infiltration.**

A) Immunohistochemical staining of MPO and citH3 in lung tissue showed reduced neutrophil infiltration in *Ninj1*^ΔNeu^ mice following LPS challenge. Scale bar = 50 μm. B) MPO activity in lung homogenates was markedly decreased in *Ninj1*^ΔNeu^ mice, *n* = 6. Data were shown as mean ± SD. ****P* < 0.001 (one‑way ANOVA followed by Tukey's post hoc test).

**Figure S8. Validation of successful HL‑60 cell differentiation by Giemsa staining.**

Giemsa staining confirms successful differentiation of HL‑60 cells into neutrophil‑like cells induced by ATRA/DMSO.

**Figure S9. Real-time PCR reveals multiple pore‑forming proteins in LPS‑primed mouse neutrophils undergoing NETosis.**

Neutrophils isolated from mice were treated with LPS (100 ng/mL) to induce NETosis. The mRNA levels of indicated pore-forming proteins, including *Gsdmd*, *Mlkl*, and *Ninj1*, were measured by Real-time PCR. *n* = 6. Data were shown as mean ± SD. ***P* < 0.01 (unpaired two‑tailed Student's *t*‑test).

**Figure S10. Time‑dependent formation of NETosis and upregulation of NINJ1 in the absence of apoptosis in PMA‑stimulated mouse neutrophils.**

A) Mouse primary neutrophils were stimulated with PMA (50 nM) for the indicated time points (0, 30, 60, 120, 150, and 180 min). Whole‑cell lysates were subjected to Western blot. β‑actin served as a loading control. B-D) Quantification of NINJ1, citH3, and caspase 3 protein levels. Data are representative of three independent experiments (*n* = 3). Data were shown as mean ± SD. **P* < 0.05, ***P* < 0.01, and****P* < 0.001 (one‑way ANOVA followed by Tukey's post hoc test).

**Figure S11. NINJ1 oligomerization is elevated in PMA-treated neutrophils.**

Bone marrow neutrophils from mice were treated with PMA (50 nM) for 3 h. A) Native-PAGE analysis of NINJ1 oligomerization in PMA-treated neutrophils. SDS-PAGE analysis of NINJ1 monomer and β-actin was used as the internal control. B) Quantification of NINJ1 monomer protein levels, *n* =3. Data were shown as mean ± SD. ***P* < 0.01 (unpaired two‑tailed Student's *t*‑test).

**Figure S12. Validation of Ninj1 knockdown and inhibition of PMA-induced NETs release upon NINJ1 silencing.**

A-B) Validation of Ninj1 knockdown efficiency by Western blot. C) Immunofluorescence images of NETs in dHL-60 transfected with either Ctrl shRNA or *Ninj1* shRNA after the PMA stimulation. Scale bar = 40 μm, *n* = 3. Data were shown as mean ± SD. ****P* < 0.001 (one‑way ANOVA followed by Tukey's post hoc test).

**Figure S13. Ninj1 knockdown prevents PMR in LPS-treated neutrophils.**

A) Native-PAGE analysis of NINJ1 oligomerization in PMA- and LPS-treated primary mouse neutrophils. SDS-PAGE analysis of NINJ1 monomer. B) Assessment of plasma membrane integrity by laser-scanning confocal microscopy in dHL-60 transfected with either Ctrl shRNA or Ninj1 shRNA after the LPS stimulation. Scale bar = 15 μm. *n* = 3.

**Figure S14. No effect of untreated K45Q on neutrophil NINJ1 oligomerization and NETs release.**

A) Native-PAGE was employed to assess NINJ1 oligomerization in neutrophils individually transfected with either NINJ1^WT^ or the NINJ1-K45Q mutant. B) Immunofluorescence images of NETs in dHL-60 transfected with either NINJ1^WT^ or the NINJ1-K45Q mutant after the PMA stimulation. Scale bar = 40 μm. *n* = 3.

**Figure S15. No alteration in NETosis formation in Ninj1‑silenced and NINJ1‑N60A mutant neutrophils.**

A) Expression of citH3, MPO, and NE was examined in neutrophils transfected with either Ctrl shRNA or Ninj1 shRNA after the PMA stimulation. B) Asparagine sites (N) were mutated to alanine (A). The presence of citH3, MPO, and NE in complete lysates was confirmed by Western blot. *n* = 3.

**Figure S16. No effect of untreated N60A on neutrophil NINJ1 oligomerization and NETs release.**

A) Native-PAGE was employed to assess NINJ1 oligomerization in neutrophils individually transfected with either NINJ1^WT^ or the NINJ1-N60A mutant. B) Immunofluorescence images of NETs in dHL-60 transfected with either NINJ1^WT^ or the NINJ1-N60A mutant after the PMA stimulation. Scale bar = 40 μm. *n* = 3.

**Figure S17. Lack of upregulated NINJ1 phosphorylation upon PMA stimulation.**

A) To predict potential phosphorylation sites, Bioinformatic analysis with CSSPalm suggested that NINJ1 contains several putative phosphorylation sites, notably at Thr18, Ser25, Ser46, and Ser50. B) Phosphoproteomic profiling of PMA-treated neutrophils identified 2303 upregulated phosphoproteins, none of which corresponded to NINJ1. C) Phosphoproteomic profiling of PMA-treated neutrophils did not detect any upregulated phosphorylation at the predicted sites on NINJ1. *n* = 3.
